# AI-Generated Microlearning for Plastic Surgery Residency: Single-Arm Pre-Post Feasibility Study

**DOI:** 10.2196/88695

**Published:** 2026-07-14

**Authors:** Marius Drysch, Sonja Verena Schmidt, Felix Reinkemeier, Flemming Puscz, Alexander Fiedler, Maria Fueth, Marcus Lehnhardt, Alexander Sogorski, Christoph Wallner

**Affiliations:** 1 Department of Plastic Surgery BG University Hospital Bergmannsheil Bochum Bochum, North Rhine-Westphalia Germany

**Keywords:** artificial intelligence, graduate medical education, plastic surgery, microlearning, large language models, feasibility studies, multiple-choice questions

## Abstract

**Background:**

Surgical residency training faces mounting pressure from expanding subspecialty knowledge requirements alongside compressed learning opportunities. Microlearning, defined as brief, focused educational episodes, offers a pedagogically sound approach suited to fragmented clinical schedules, while large language models present capabilities for scalable content generation. However, the feasibility, quality, and educational value of large language model–generated microlearning in surgical training remain unexplored.

**Objective:**

This study aimed to assess the feasibility and acceptability of artificial intelligence (AI)-generated microlearning modules among plastic surgery residents, evaluate content quality through systematic faculty evaluation, and generate preliminary effect size estimates for self-perceived knowledge and confidence changes.

**Methods:**

A single-arm pilot feasibility study with a pre-post design was conducted at an academic plastic surgery residency program (N=11 residents) over 12 weeks. Six modules covering core subspecialties were developed using Google Gemini 2.5 Pro. From 60 AI-generated multiple-choice questions, 42 were selected following independent evaluation by 2 board-certified plastic surgeons using a 5-dimension rubric (accuracy, relevance, clarity, pedagogical value, and safety). Modules were released biweekly via Google Forms. Outcomes included retention, acceptability (7-point Likert scales), faculty evaluation ratings, item-level psychometrics, and pre-post changes in self-perceived knowledge and confidence. Composite scores were calculated as the mean change across all 6 subspecialty topics. Wilcoxon signed-rank tests served as the primary analysis for matched pairs (n=9). Analyses were conducted using Python 3.11.

**Results:**

All 11 residents completed postassessments. Acceptability was high across all 6 subitems (all medians ≥5 out of 7). Faculty evaluation confirmed high content quality (mean quality index 4.68/5); interrater reliability was poor (intraclass correlation coefficient (3,1)=0.231). Among 9 matched pairs, composite confidence improved significantly (median change 0.33; *d*=0.65; *P*=.047), while composite knowledge showed a medium effect (*d*=0.71; *P*=.09). Topic-specific gains were largest where baseline was lowest: hand surgery knowledge (*d*=1.48; *P*=.02) and confidence (*d*=1.41; *P*=.02). Resource use and sense-of-community measures remained stable.

**Conclusions:**

AI-generated microlearning modules are feasible and acceptable for plastic surgery residency education. Faculty-evaluated content achieved high quality ratings (mean 4.68/5), and residents showed strong engagement with preliminary improvements in self-perceived confidence. The intervention was integrated into existing learning ecosystems without disrupting traditional resources or peer dynamics. These findings provide effect size estimates to support the design of adequately powered randomized trials.

## Introduction

Surgical residency training faces growing tensions between expanding knowledge requirements and compressed learning opportunities [1,2]. Duty-hour restrictions, increasing clinical demands, and the shift toward competency-based medical education have collectively reduced protected didactic time while raising expectations for subspecialty knowledge acquisition [1,3,4]. In plastic surgery, trainees must achieve proficiency across diverse domains, from microsurgical reconstruction and hand surgery to burns, aesthetic procedures, and oncologic principles, within a finite training period [5-7]. Traditional educational models relying on scheduled lectures and self-directed textbook study struggle to accommodate the fragmented schedules characteristic of modern surgical training [8].

Microlearning, defined as brief, focused learning episodes delivered in digestible segments, offers a pedagogically sound approach to these constraints [9]. The format aligns with cognitive load theory, reducing excessive cognitive load through brief, focused content delivery [9,10]. Short modules can be completed during brief intervals between cases or during call nights without requiring sustained blocks of protected time. Multiple-choice questions (MCQs) with detailed explanations represent an effective microlearning modality, combining active retrieval practice with immediate feedback [11-13].

Concurrently, large language models (LLMs) have demonstrated the ability to generate medical MCQs that approximate the quality of expert-authored items [14-16], with specialty-specific investigations confirming acceptable psychometric properties for LLM-generated MCQs in radiology [17] and urology [18]. A randomized trial in orthopedic education further reported knowledge gains when students used an LLM as a study aid [19]. However, a systematic review noted that most work has assessed content quality rather than educational deployment [20]. No study has embedded artificial intelligence (AI)–generated MCQs within a structured microlearning curriculum for surgical residency training, leaving questions of feasibility, content safety, and efficacy in this high-stakes educational context unanswered [21,22].

To address these gaps, we conducted a pilot feasibility study implementing AI-generated microlearning modules covering 6 core plastic surgery subspecialties within an academic residency program. Module topics comprised the 4 classical pillars of the field (reconstructive surgery, aesthetic surgery, hand surgery, and burn surgery) complemented by soft tissue tumor surgery [23] and an integrative module on core surgical principles. Our primary objectives were to (1) assess the feasibility and acceptability of an LLM-generated microlearning intervention among plastic surgery residents, (2) evaluate the quality of AI-generated content through systematic expert faculty evaluation, and (3) generate preliminary effect size estimates for self-perceived knowledge and confidence changes to inform future trials. Secondary objectives examined whether the intervention integrated into existing learning ecosystems without disrupting study habits or peer learning dynamics.

## Methods

### Study Design and Setting

**Figure 1:**
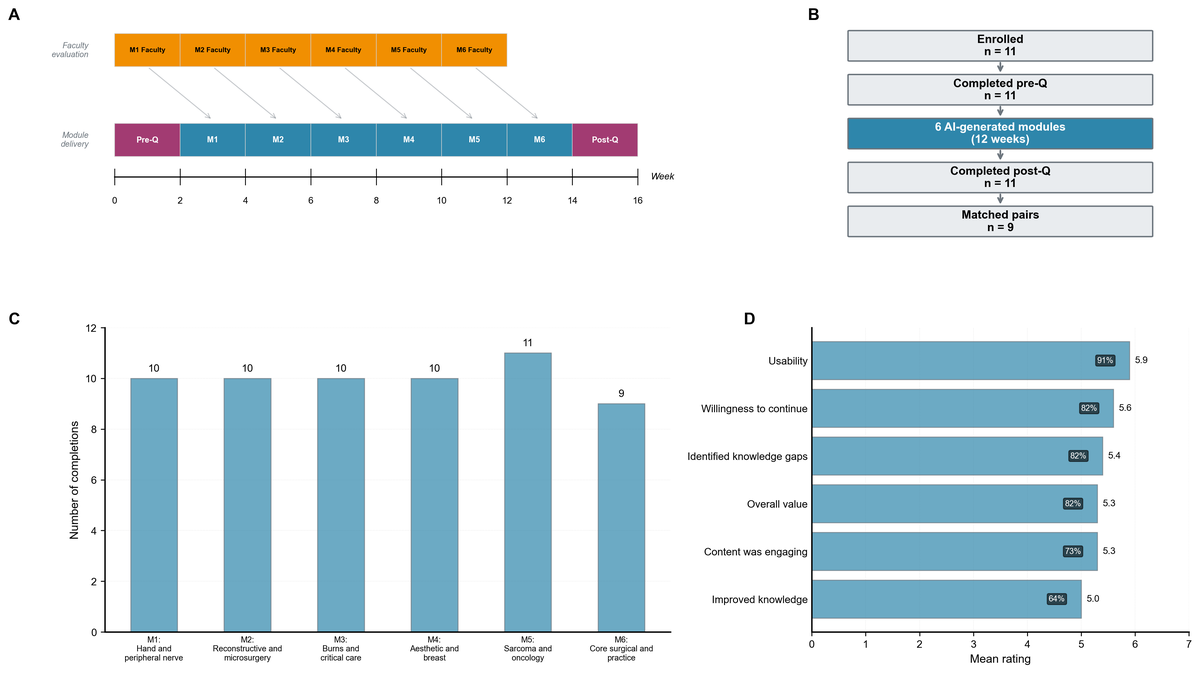
Pilot study design and feasibility outcomes. (A) Intervention timeline illustrating the rolling production model, where faculty evaluation (orange bars) occurred concurrently with the biweekly delivery of active modules (blue bars) over the 12-week period. (B) Participant flow diagram showing enrollment (n=11), postassessment completion (n=11), and the final matched cohort (n=9) used for paired analyses. (C) Module completion frequency by subspecialty topic. Bars indicate the number of module submissions per topic. (D) Postintervention acceptability ratings on a 7-point Likert scale (1=strongly disagree, 7=strongly agree). Numbers to the right of bars indicate mean ratings. Percentages inside bars indicate the proportion of residents scoring 5 or higher. AI: artificial intelligence.

A single-arm pilot feasibility study with a pre-post design was conducted at the Department of Plastic and Reconstructive Surgery, University Hospital BG Bergmannsheil Bochum, an academic medical center with an accredited plastic surgery residency program in Bochum, Germany. The study spanned a 12-week intervention period from July through September 2025. Reporting was guided by relevant elements of the CONSORT (Consolidated Standards of Reporting Trials) extension for pilot and feasibility studies [24]. The intervention followed a rolling production model in which faculty evaluation and selection of subsequent modules occurred concurrently with the biweekly delivery of active modules to residents, ensuring continuous content delivery throughout the study period (Figure 1A).

### Participants

All residents enrolled in the plastic surgery residency program were eligible for participation using a census sampling approach (N=11). No a priori sample size calculation was performed, consistent with the pilot feasibility design; the sample comprised the entire residency cohort to maximize enrollment and provide preliminary data across training levels.

All residents actively enrolled in the plastic surgery residency program at any postgraduate year (PGY) level were eligible; no exclusion criteria were applied.

The 11 enrolled residents were distributed across junior (PGY 1-2; n=4), mid-level (PGY 3-4; n=2), and senior (PGY 5-6; n=5) training years. Participation was voluntary, and no incentives were provided beyond access to the educational content. All 11 participants completed both pre- and postintervention assessments. Two postassessments could not be linked to their corresponding pre-assessments due to inconsistent pseudonym entry, yielding 9 matched pairs for paired analyses (Figure 1B).

### AI-Generated Microlearning Modules

#### Module Topics

Six microlearning modules were developed covering core plastic surgery subspecialties: (1) hand and peripheral nerve surgery, (2) reconstructive principles and microsurgery, (3) burns and surgical critical care, (4) aesthetic and breast surgery, (5) sarcoma and oncologic surgery, and (6) core surgical and practice management. Topics were selected to align with the German plastic surgery curriculum (from the Accreditation Council for Graduate Medical Education, and the European Board of Plastic Reconstructive and Aesthetic Surgery; *Musterweiterbildungsordnung* [MWBO] in German) and to broadly correspond to international standards, such as those of the Accreditation Council for Graduate Medical Education and the European Board of Plastic Reconstructive and Aesthetic Surgery [25,26].

#### Item Generation

MCQs were generated using Google Gemini 2.5 Pro (Google; the first module used Gemini 2.5 Pro Preview, subsequent modules used the stable release). For each module, 10 initial questions were generated in separate LLM sessions using a standardized prompt (Multimedia Appendix 1). The prompt instructed the model to emulate board-style item writing as found in relevant standard textbooks (eg, *Essentials of Plastic Surgery*), target the Bloom’s taxonomy “application” level or higher, adhere to evidence-based accuracy and patient safety principles, and integrate systematic knowledge such as clinical classification systems and pertinent anatomy. Each generated item was required to include a clinical vignette (2 to 4 sentences) or direct question stem, five answer options with plausible distractors, identification of the correct answer, and a structured explanation comprising: (1) rationale for the correct answer, (2) rationales for each incorrect option, (3) a systematic knowledge highlight identifying the core concept, and (4) broader educational links contextualizing the topic within plastic surgery training.

#### Item Selection

From the initial pool of 60 items (10 per module), 42 questions (7 per module) were selected for the pilot. Following independent evaluation (see the Faculty Quality Evaluation Protocol section), both faculty reviewers jointly reviewed the aggregated ratings and discussed each item, considering overall quality scores, content coverage across subtopics within each module, and alignment with residency training objectives. Items with lower ratings were retained when they addressed essential subtopics not covered by higher-scoring alternatives. Both reviewers reached consensus on all inclusion and exclusion decisions. The quality distributions of selected and excluded items are compared in Figure S3 in Multimedia Appendix 2.

#### Delivery

Modules were delivered via Google Forms, accessible on desktop and mobile devices. Residents received email and messenger notifications when new modules became available, released on a biweekly schedule (1 module every 2 weeks). Deadlines were set to the end of the study period, allowing self-paced engagement rather than enforcing a fixed completion window per module.

### Faculty Quality Evaluation Protocol

All AI-generated content was independently evaluated by 2 attending plastic surgeons, each with more than 10 years of clinical experience and board certification in plastic surgery. Both faculty members were blinded to each other’s assessments during independent evaluation. To ensure standardization, faculty were provided with a written grading guideline outlining the evaluation rubric, scoring anchors, and examples of acceptable versus unacceptable item features. Faculty evaluated each question and its accompanying explanation across five dimensions using standardized 5-point scales (1=poor, 5=excellent):

1. Accuracy and currency—factual correctness, alignment with current evidence-based practice guidelines, and absence of outdated information.
2. Relevance and objective alignment—clinical relevance to plastic surgery training and alignment with core competency frameworks (from the MWBO).
3. Clarity and quality of construction—question and explanation clarity, absence of ambiguity, quality of distractors, and logical organization.
4. Pedagogical value—educational effectiveness for the target learner level, appropriate difficulty calibration, and comprehensive answer justification.
5. Ethical and safety considerations—freedom from demographic or cultural biases, appropriate safety cautions, and absence of potentially harmful recommendations.

The overall quality index for each item was calculated as the mean across all 5 dimensions. The complete rubric with detailed scoring criteria is provided in Multimedia Appendix 1. Faculty reviewed all 60 items in a structured, module-by-module sequence before pilot implementation. Interrater reliability was assessed using intraclass correlation coefficients (ICC; two-way mixed-effects model, single measures, ICC[3,1]) [27].

### Pre- and Postintervention Assessments

Prior to module release, all residents completed a baseline questionnaire. Following the 12-week intervention, residents completed an identical questionnaire with additional post-only measures. Table 1 lists all questionnaire items with verbatim wording, response formats, and assessment timing.

**Table 1:** Pre- and postintervention questionnaire items.

Item	Domain	Item wording	Response format	Timing
1	Demographics	Postgraduate year (PGY) level in plastic surgery	Single choice: PGY-1/2; PGY-3/4; PGY-5/6; Prefer not to disclose	Pre + post
2	Knowledge	“My current *knowledge* of this topic is:”^a^	7-point scale (1=very low to 7=very high); matrix with 6 subspecialty topics^b^	Pre + post
3	Confidence	“My current *confidence* in clinically applying knowledge related to this topic is:”^a^	7-point scale (1=very low to 7=very high); matrix with 6 subspecialty topics^b^	Pre + post
4	Study habits	“On average, how many hours per week do you dedicate to independent study specifically for Plastic Surgery topics?”	Hours per week (0 to >6 in 0.5-hour increments)	Pre + post
5	Study habits	“How often do you use the following resources?”^a^	5-point frequency scale (daily, weekly, monthly, rarely, never); matrix with 6 resource types^c^	Pre + post
6	Study habits	“If you use any important study resources not listed in the previous question, please specify them here.”	Open text	Pre + post
7	Study habits	“I actively seek out new information or resources to deepen my understanding of complex plastic surgery topics.”	7-point Likert (1=strongly disagree to 7=strongly agree)	Pre + post
8	Study habits	“How would you rate your familiarity with using AI^d^ tools (eg, ChatGPT) for studying plastic surgery topics?”	7-point scale (1=very low to 7 = very high)	Pre + post
9	Study habits	“What are the primary barriers that limit your ability to study independently?”	Multiselect checklist (9 predefined options + Other)^e^	Pre + post
10	Community	“I feel that our residency program effectively addresses important learning needs that are common among Plastic Surgery trainees like myself.”	7-point Likert (1=strongly disagree to 7=strongly agree)	Pre + post
11	Community	“I often discuss challenging clinical topics or new learnings with my fellow residents.”	7-point Likert (1=strongly disagree to 7=strongly agree)	Pre + post
12	Community	“I feel a sense of connection with my fellow residents through our shared learning experience in plastic surgery.”	7-point Likert (1=strongly disagree to 7=strongly agree)	Pre + post
13	Community	“I feel comfortable asking my fellow residents for help or admitting when I don’t know something.”	7-point Likert (1=strongly disagree to 7=strongly agree)	Pre + post
14	Community	“In our program, residents actively share knowledge and opportunities to help each other succeed.”	7-point Likert (1=strongly disagree to 7=strongly agree)	Pre + post
15	Participation	“How many of the six modules did you complete?”	Single choice: 1 through 6	Post only
16	Participation	“How carefully did you engage with the module content?”	5-point ordinal scale^f^	Post only
17	Participation	“On average, how much time did you spend on each module?”	Categorical: <10 min; 10-20 min; 20-40 min; 40-60 min; >60 min	Post only
18	Acceptability	“This program was a valuable addition to my residency education.”	7-point Likert (1=strongly disagree to 7=strongly agree)	Post only
19	Acceptability	“The AI-generated MCQs^g^ helped me effectively identify my knowledge gaps.”	7-point Likert (1=strongly disagree to 7=strongly agree)	Post only
20	Acceptability	“Participating in this pilot project has improved my knowledge base in the topics covered.”	7-point Likert (1=strongly disagree to 7=strongly agree)	Post only
21	Acceptability	“The micro-learning format was an engaging way to study complex topics.”	7-point Likert (1=strongly disagree to 7=strongly agree)	Post only
22	Acceptability	“The program’s format was user-friendly and easy to navigate.”	7-point Likert (1=strongly disagree to 7=strongly agree)	Post only
23	Open feedback	“What did you like most about this program?”	Open text	Post only
24	Open feedback	“What suggestions do you have for future iterations of this micro-learning format?”	Open text	Post only
25	Acceptability	“If this program were to continue, would you like to participate again?”	7-point scale (1=definitely not to 7=definitely yes)	Post only

^a^Matrix item: the stem was rated once for each row listed in the corresponding footnote.

^b^Subspecialty topics (rated as separate rows within one matrix): (1) hand and peripheral nerve surgery, (2) reconstructive and microsurgery, (3) burns and surgical critical care, (4) aesthetic and breast surgery, (5) sarcoma and oncologic surgery, (6) core surgical and practice management.

^c^Resource types (rated as separate rows within one matrix): (1) standard plastic surgery textbooks (eg, Essentials of Plastic Surgery), (2) journal articles (eg, Plastic and Reconstructive Surgery), (3) departmental resources, (4) online video platforms (eg, YouTube), (5) online databases (eg, Amboss, Microsurgeon), (6) AI-powered tools (eg, ChatGPT and Gemini).

^d^AI: artificial intelligence.

^e^Predefined barrier options: Limited time due to clinical duties; Fatigue or mental exhaustion from clinical work; Lack of a clear study plan or curriculum; Limited access to high-quality study materials; Financial constraints related to educational resources; Distracting study environment or difficulty maintaining focus; Lack of motivation or feelings of burnout; Family or personal commitments; Preference for hands-on learning.

^f^Content engagement response options: (1) I answered the questions quickly, (2) I answered the questions without deep reflection, (3) I answered the questions and reflected on the explanations, (4) I answered, reflected, and sometimes looked up related topics, (5) I answered, reflected deeply, and frequently consulted additional sources.

^g^MCQ: multiple-choice question.

### Operational Definitions

Knowledge change was operationalized as the difference between postintervention and pre-intervention self-reported subspecialty knowledge scores on the 7-point Likert scale. Composite knowledge change was calculated as the mean of individual change scores across all 6 subspecialty topics. Confidence change was calculated identically for the confidence domain.

Explanation quality was operationalized as the mean faculty evaluation score across the 5 quality dimensions for the explanation component of each item, as rated independently by both evaluators. This metric was used for correlation analyses examining the relationship between content quality and learning outcomes at the module level.

### Outcomes

Outcomes were organized into a hierarchical framework reflecting the pilot nature of this study.

#### Primary Outcomes (Feasibility)

Retention rate (proportion completing postintervention assessment), module completion rates, time spent (minutes per module and cumulative time across all modules), content engagement level, and acceptability ratings.

#### Secondary Outcomes (Content Quality)

Faculty evaluation ratings across the 5 quality dimensions and the overall quality index, interrater reliability (ICC), and item-level psychometric properties (item difficulty and item discrimination).

#### Exploratory Outcomes (Educational Efficacy)

Knowledge and confidence change scores, reported as both composite measures (mean across all 6 topics) and topic-specific measures for each subspecialty. Given the pilot sample size (n=9 matched pairs), these analyses were hypothesis-generating rather than confirmatory.

#### Exploratory Outcomes (Integration)

Changes in study habits (weekly study time and resource use patterns), AI tool familiarity, and sense of community dimensions. These analyses tested the preliminary hypothesis that asynchronous AI-based microlearning would integrate into existing learning structures without disrupting social learning dynamics or increasing perceived workload.

### Statistical Analysis

As a pilot feasibility study designed to assess implementation feasibility and generate effect size estimates for future trials, formal a priori power calculations were not conducted. The sample included all eligible program residents (N=11).

#### Descriptive Statistics

Feasibility metrics were reported using proportions with 95% CIs for categorical outcomes. For ordinal outcomes (Likert-scale responses), medians (IQRs) were reported as the primary summary measure. Means (SDs) are reported where appropriate for comparison with existing literature. Faculty evaluation ratings were summarized using mean (SDs) and 95% CIs.

#### Primary Analysis

Pre-post changes in educational and integration outcomes were analyzed using Wilcoxon signed-rank tests for participants with complete matched data (n=9 matched pairs). Nonparametric testing was selected as the primary analysis given the ordinal nature of Likert-scale responses and the small sample size, where parametric assumptions may be violated. Normality of difference scores was assessed using Shapiro-Wilk tests.

#### Effect Sizes

Cohen *d* was calculated for descriptive comparison of pre-post changes, interpreted as small (*d*=0.2), medium (*d*=0.5), or large (*d*=0.8) effects [28]. CIs for Cohen *d* were derived from the t-distribution; as these are parametric approximations, minor discrepancies with Wilcoxon-based inference are expected for small samples. Rank-biserial correlations (r) were calculated as nonparametric effect size measures for Wilcoxon signed-rank tests.

#### Correlation Analyses

Associations between module-level outcomes (MCQ accuracy, perceived difficulty, quality, and relevance) and educational outcomes (knowledge change) were examined using Pearson correlation coefficients.

#### Item-Level Psychometrics

Item difficulty was calculated as the proportion of correct responses (*P* value). Item discrimination was assessed using point-biserial correlations between item scores and total test scores. Internal consistency was evaluated using Cronbach ɑ at the module level. The

ɑ level was set at *P*<.05 (2-tailed). Given the pilot nature and hypothesis-generating purpose, no adjustments for multiple comparisons were applied to exploratory analyses. Analyses with varying sample sizes due to item-level missing data report n per analysis. All analyses were conducted using Python (version 3.11) with *scipy*, *pandas*, *statsmodels*, *matplotlib*, and *seaborn* libraries. Complete statistical analysis code is available upon request.

### Ethical Considerations

This study was reviewed by the Ethics Committee of the Medical Chamber of Westfalen-Lippe (Ärztekammer Westfalen-Lippe). The committee confirmed that formal ethical approval was not required, as the study exclusively involved anonymized data and therefore posed no risk to participant rights or safety, in accordance with the Declaration of Helsinki [29] and section 15 of the Professional Code of Conduct (reference 2025-605-f-N). All participants provided informed consent.

## Results

### Participant Characteristics and Study Flow

All 11 residents enrolled in the plastic surgery residency program consented to participate and completed both baseline and postintervention assessments. The cohort comprised junior (PGY 1-2; n=4), mid-level (PGY 3-4; n=2), and senior (PGY 5-6; n=5) trainees. Two postassessments could not be linked to their corresponding preassessments due to inconsistent pseudonym entry, yielding 9 matched pairs for paired analyses (Figure 1B). Module completion ranged from 9 to 11 submissions across the 6 modules, with modules 1-4 completed by 10 residents (91%), module 5 by 11 (100%), and module 6 by 9 (82%) (Figure 1C). Estimated cumulative time spent was 60 to 120 minutes over 12 weeks, equivalent to 10 to 20 minutes per module and comparable to 1-2 traditional didactic sessions.

### Feasibility and Acceptability

Content engagement was moderate to high: 8 of 11 residents (73%) reported answering questions and reflecting on explanations, and 2 (18%) reported additionally consulting related topics (Table S1 in Multimedia Appendix 3). Postintervention acceptability ratings were uniformly positive, with all 6 items exceeding the scale midpoint of 4 on the 7-point scale (1 = strongly disagree, 7 = strongly agree) (Figure 1D). Usability received the highest rating (median 6, IQR 6-6; 10/11 scored 5 or higher). Willingness to continue was similarly high (median 6, IQR 5-7; 9/11 scored 5 or higher). Knowledge gap identification and overall training value each had 9 of 11 residents scoring 5 or higher. Engagement quality had a median of 6 (IQR 4.5-6.0; 8/11 scored 5 or higher). Perceived knowledge improvement received the lowest acceptability rating (median 5, IQR 4-6; 7/11 scored 5 or higher). Item-level response distributions are provided in Table S1 in Multimedia Appendix 3.

### Content Quality and Expert Evaluation

**Figure 2:**
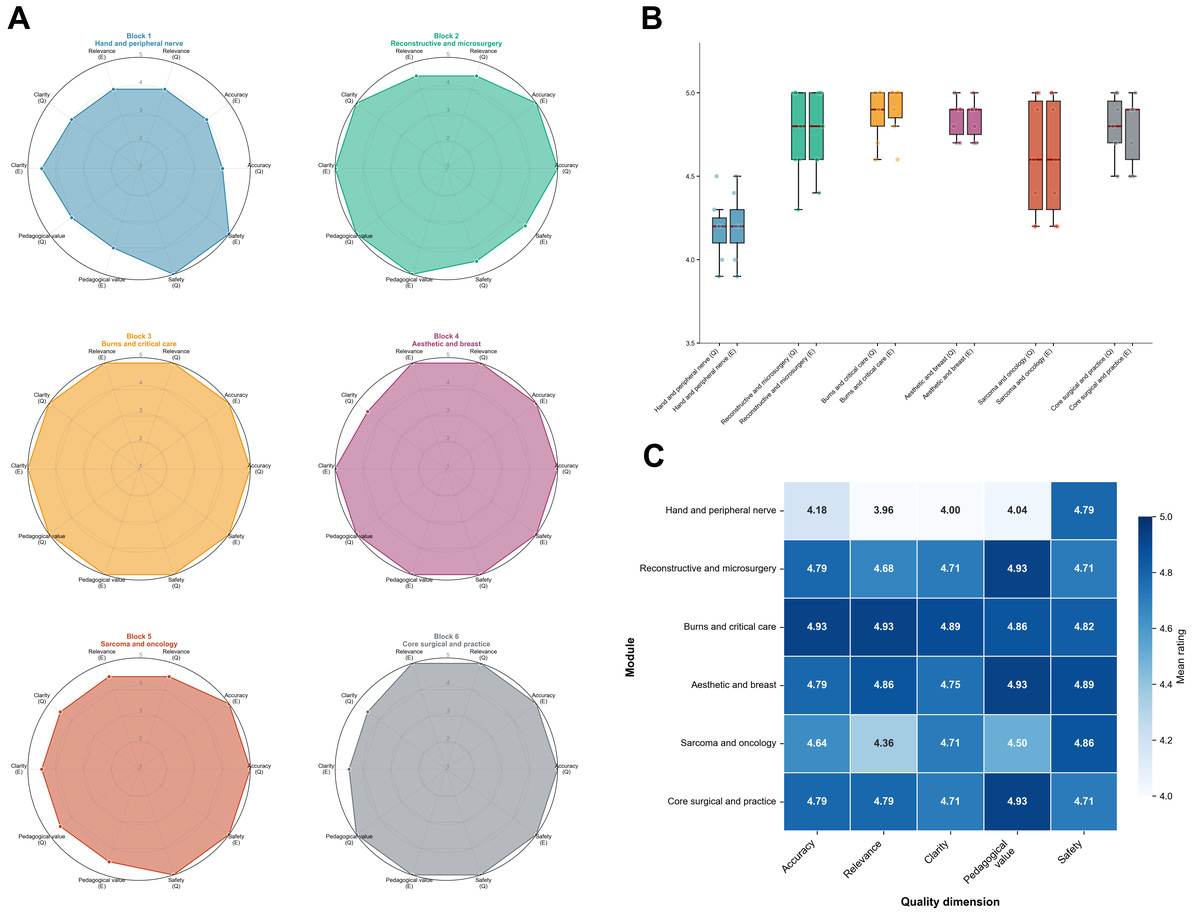
Faculty quality evaluation of artificial intelligence-generated items selected for the pilot (n = 42). (A) Radar plots displaying separate mean quality ratings for questions (Q) and explanations (E) per subspecialty module across five dimensions (Accuracy, Relevance, Clarity, Pedagogical Value, Safety). (B) Box plots showing the distribution of mean scores for Questions (Q) and Explanations (E). Center lines represent medians, box limits indicate the interquartile range, and whiskers represent the range. (C) Heatmap of mean ratings for each quality dimension across modules. Darker shading indicates higher ratings.

Of the 60 AI-generated items evaluated by 2 board-certified faculty members, 42 were selected for the pilot (7 per module). The overall quality index for selected items was 4.68 out of 5 (SD 0.44), compared with 4.28 out of 5 for excluded items (Figure S3 in Multimedia Appendix 2). Question and explanation quality showed strong positive correlations across dimensions (pedagogical value *r*=0.94, accuracy *r*=0.91; Figure S2 in Multimedia Appendix 2), while the safety dimension showed negligible correlation with other metrics (*r*=–0.05 with relevance), indicating that clinical safety standards were maintained independent of variation in other quality dimensions. Score distributions for questions and explanations showed close alignment within each subspecialty module (Figure 2B). Among the 5 evaluation dimensions, safety received the highest mean rating (4.80), followed by pedagogical value (4.70), accuracy (4.68), clarity (4.63), and relevance (4.60) (Figure 2C). The Safety dimension remained consistently high across all modules (range 4.71-4.89), independent of variation in other dimensions. Module-level quality varied, with burns and critical care scoring highest (4.89) and hand and peripheral nerve surgery scoring lowest (4.19; notably, this was the only module generated with Gemini 2.5 Pro Preview rather than the stable release) (Figure 2A). Individual item ratings are provided in Table S2 in Multimedia Appendix 3. Faculty quality ratings for all 60 generated items and interdimensional correlations are shown in Figures S1 and S2 in Multimedia Appendix 2.

Interrater reliability for the overall quality evaluation was poor (ICC(3,1)=0.231, 95% CI 0.154-0.305; *P*<.001). Among individual dimensions, pedagogical value showed the highest agreement (ICC=0.314, 95% CI 0.143-0.466) and relevance the lowest (ICC=0.180, 95% CI 0.002-0.348). The low agreement is consistent with the subjective nature of content quality judgments between 2 independent raters and the limited precision afforded by a 2-rater design.

### Item Characteristics and Module Performance

**Figure 3:**
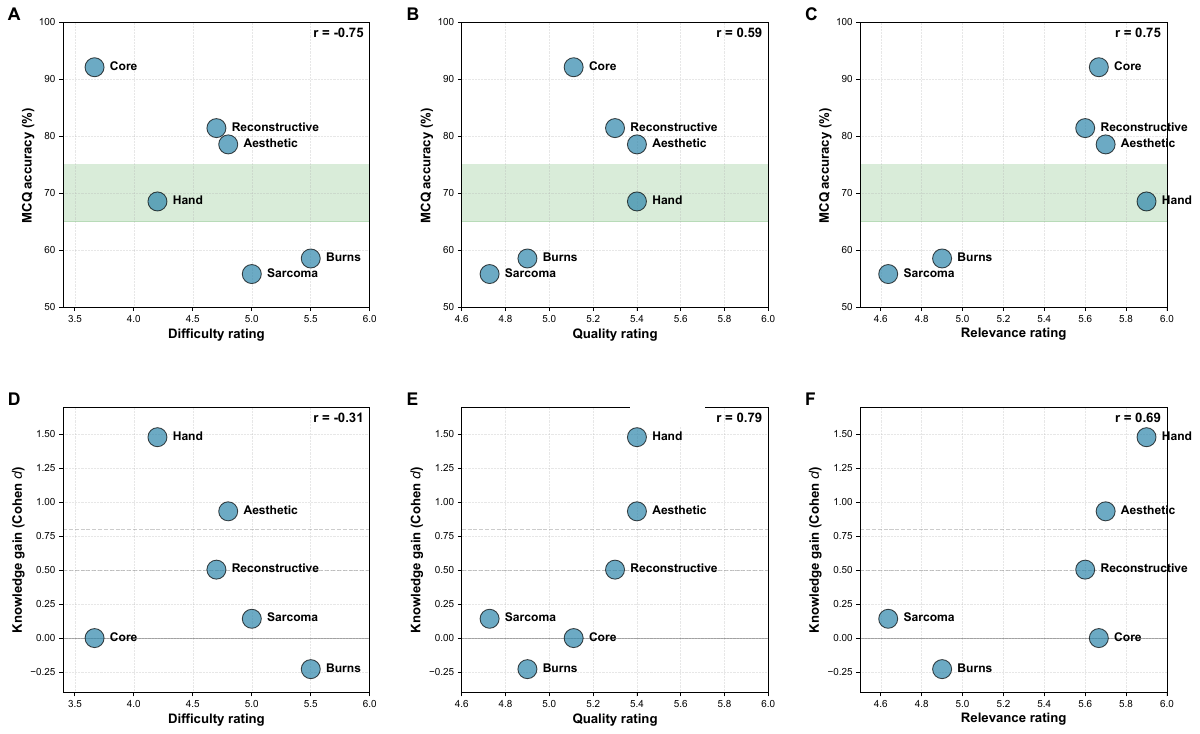
Association between resident perceptions and performance outcomes. (A-C) Scatter plots correlating mean multiple-choice question accuracy (%) with resident ratings of module difficulty (A), quality (B), and relevance (C). The shaded region represents the recommended optimal difficulty zone (65%-75%) for formative assessment. (D-F) Scatter plots correlating knowledge change (Cohen d) with resident ratings of module difficulty (D), quality (E), and relevance (F). The horizontal dotted lines indicate the thresholds for medium (d=0.5) and large (d=0.8) effect sizes. Pearson correlation coefficients (r) are displayed for each comparison. MCQ: multiple-choice questions.

Overall person-level accuracy across the 42 selected items averaged 72.5% (SD 14.1%), falling within the recommended 65% to 75% zone for formative assessment (Figure 3A-C). Module-level accuracy ranged from 55.8% (sarcoma and oncology) to 92.1% (core surgical and practice) (Table 2).

Item-level psychometric properties varied across modules (Table 2). Mean item difficulty ranged from 0.56 (sarcoma and oncology) to 0.92 (core surgical and practice). mean item discrimination (point-biserial correlation) ranged from -0.34 (core surgical and practice) to 0.46 (burns and critical care; sarcoma and oncology). Internal consistency was low overall, with 3 of 6 modules yielding negative Cronbach ɑ values (–0.82 to –0.20). Each module’s 7 items spanned distinct subtopics rather than targeting a single construct, which likely accounts for this pattern. Burns (ɑ=0.69) and sarcoma (ɑ=0.74) were the only modules with positive internal consistency, and both also had the highest item discrimination.

At the module level, perceived difficulty showed a strong negative association with MCQ accuracy (*r*=–0.75; Figure 3A), indicating that modules rated as more challenging yielded lower scores. Perceived relevance (*r*=0.75; Figure 3C) and content quality (*r*=0.59; Figure 3B) showed positive associations with accuracy. Content quality correlated most strongly with knowledge change (*r*=0.79; Figure 3E), followed by perceived relevance (*r*=0.69; Figure 3F), while difficulty showed only a weak negative association (*r*=–0.31; Figure 3D). Conversely, core surgical and practice showed the highest accuracy (92.1%) but no knowledge change (*d*=0), suggesting a ceiling effect. Module-level perception outcomes and the complete correlation matrix are provided in Table S3 in Multimedia Appendix 3.

**Table 2:** Module performance and item-level psychometric properties.

Modules	n	MCQ^a^ accuracy, % median (IQR)	Mean item difficulty (*p*)^b^	Mean item discrimination (*r*_pb_)	Cronbach ɑ
Hand and peripheral nerve^c^	10	71.4 (60.7-71.4)	0.69	–0.12	–0.37
Reconstructive and microsurgery	10	85.7 (71.4-85.7)	0.81	–0.04	–0.20
Burns and critical care	10	50.0 (42.9-82.1)	0.59	0.46	0.69
Aesthetic and breast	10	78.6 (71.4-85.7)	0.79	0.07	0.09
Sarcoma and oncology	11	42.9 (28.6-85.7)	0.56	0.46	0.74
Core surgical and practice	9	85.7 (85.7-100)	0.92	–0.34	–0.82
Overall	10^d^	71.4 (57.1-85.7)	0.73	0.08	—^e^

^a^MCQ: multiple-choice question.

^b^Item difficulty (*p*) = proportion of correct responses; values approaching 0.50 indicate maximum discriminating power, values approaching 1.00 indicate easy items. Item discrimination (*r*_pb_) = point-biserial correlation between item score and total module score. Cronbach ɑ assesses internal consistency at the module level; negative values for three modules reflect the intentionally heterogeneous content within each module, where 7 items were designed to span distinct subtopics rather than measure a single construct. Individual item statistics are provided in the supplementary materials.

^c^Generated with Gemini 2.5 Pro Preview; all other modules used the stable release.

^d^Median module completion.

^e^Not applicable.

### Educational Efficacy

**Figure 4:**
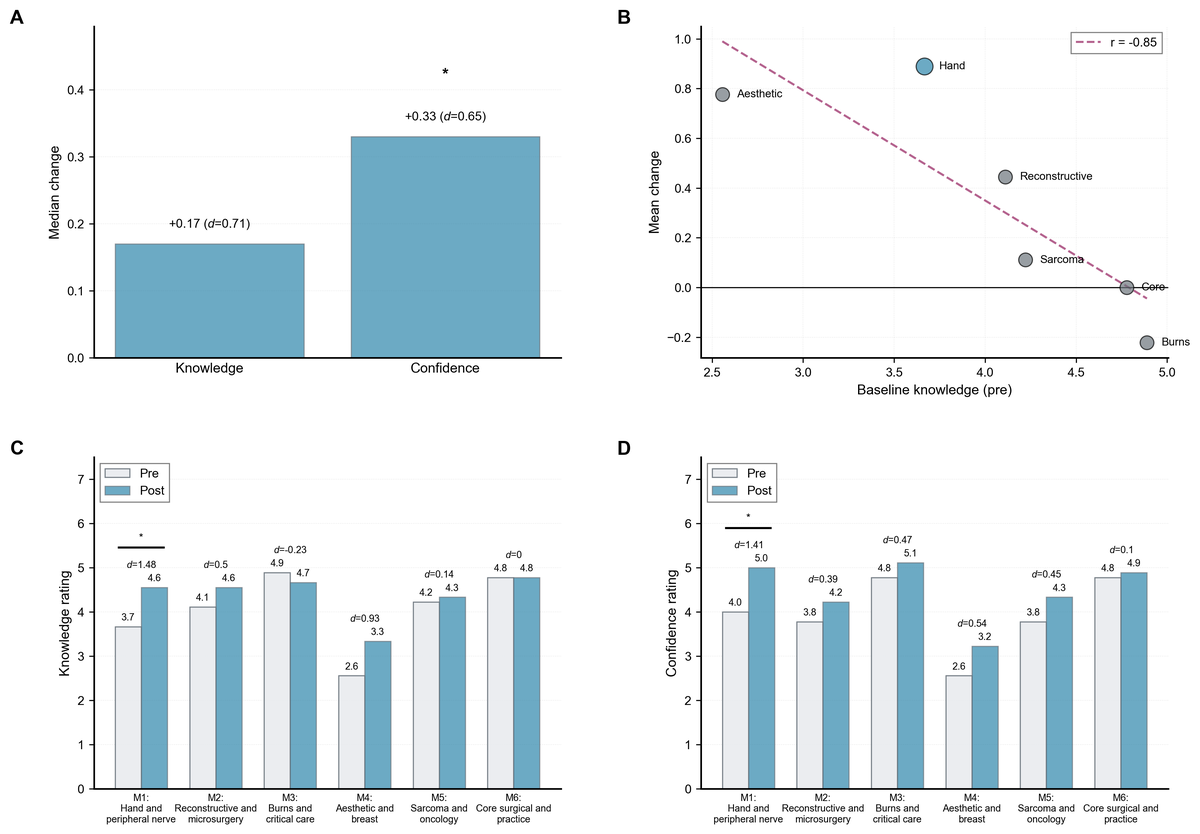
Educational efficacy outcomes. (A) Composite change in self-reported knowledge and confidence scores (n = 9 matched pairs), displaying the median change and effect sizes (Cohen d). *P<.05 by Wilcoxon signed-rank test. (B) Relationship between baseline knowledge and knowledge change across modules (r=–0.85), indicating that topics with lower starting scores showed greater improvement. (C) Pre- and postintervention self-perceived knowledge by subspecialty module. (D) Pre- and postintervention self-perceived confidence by subspecialty module. Cohen d values indicate the effect size for each comparison; *P<.05 by Wilcoxon signed-rank test.

Among the 9 matched pairs, composite self-perceived confidence improved significantly (median change 0.33; *d*=0.65; *P*=.047), while composite knowledge showed a medium effect that did not reach statistical significance (median change 0.17; *d*=0.71; *P*=.09) (Figure 4A; Table 3).

Topic-specific outcomes are summarized in Table 3. The largest effects emerged in hand and peripheral nerve surgery, with significant improvements in both knowledge (*d*=1.48; *P*=.02) and confidence (*d*=1.41; *P*=.02). Aesthetic and breast surgery showed a large knowledge effect size (*d*=0.93) that did not reach significance (*P*=.06). Burns and critical care was the only module with a negative knowledge trajectory (*d*=–0.23), while core surgical and practice showed no knowledge change (*d*=0). A consistent inverse relationship between baseline scores and improvement was observed across modules (*r*=–0.85; Figure 4B), with the largest gains occurring in topics with the lowest baseline self-assessments.

A forest plot of all effect sizes is available in Figure S4 in Multimedia Appendix 2. Complete parametric statistics are provided in Table S4 in Multimedia Appendix 3 for methodological transparency.

**Table 3:** Pre-post changes in self-perceived knowledge and confidence (n=9 matched pairs).

Outcomes		Pre median (IQR)	Post median (IQR)	Median diff	*W*	*P* value	*r*	*d* (95% CI)
**Composite measures^a^**								
	Knowledge	4 (3.33-4.33)	4.17 (4.00-4.83)	0.17	7.5	.09	0.67	0.71 (–0.06 to 1.48)
	Confidence	4.17 (3-4.33)	4.67 (4.17-5.17)	0.33	5.5	.047	0.76	0.65 (–0.11 to 1.42)
**Topic-specific: knowledge**								
	Hand and peripheral nerve	4 (3-4)	5 (4-5)	1	1.5	.02	0.93	1.48 (0.71-2.25)
	Reconstructive and microsurgery	4 (3-5)	5 (4-5)	0	11.5	.31	0.49	0.50 (–0.26 to 1.27)
	Burns and critical care	4 (4-6)	4 (4-6)	0	18	.75	0.20	–0.23 (–1 to 0.54)
	Aesthetic and breast	2 (2-3)	4 (3-4)	1	5	.06	0.78	0.93 (0.16-1.70)
	Sarcoma and oncology	4 (4-4)	4 (4-5)	0	19	≥.99	0.16	0.14 (–0.63 to 0.91)
	Core surgical and practice	5 (4-6)	5 (4-5)	0	20	.94	0.11	0 (–0.77 to 0.77)
**Topic-specific: confidence**								
	Hand and peripheral nerve	4 (4-5)	5 (4-6)	1	1.5	.02	0.93	1.41 (0.65-2.18)
	Reconstructive and microsurgery	4 (2-5)	5 (3-5)	0	14.5	.50	0.36	0.39 (–0.38 to 1.16)
	Burns and critical care	5 (4-5)	5 (4-6)	0	12	.38	0.47	0.47 (–0.30 to 1.24)
	Aesthetic and breast	2 (2-3)	3 (3-4)	0	11	.25	0.51	0.54 (–0.22 to 1.31)
	Sarcoma and oncology	4 (3-4)	5 (3-5)	1	12.5	.36	0.44	0.45 (–0.32 to 1.22)
	Core surgical and practice	5 (4-6)	5 (5-5)	0	19	≥.99	0.16	0.11 (–0.66 to 0.87)

^a^Bold rows indicate statistical significance (*P*<.05). Medians and IQR are reported on the original 7-point Likert scale (1=strongly disagree/not at all confident to 7=strongly agree/very confident). Median diff=median of individual paired differences. *W*=Wilcoxon signed-rank test statistic (smaller values indicate greater positive shift); *r*=rank-biserial correlation (nonparametric effect size); *d* = Cohen *d* with 95% CI. Complete parametric statistics are provided in Table S4 in Multimedia Appendix 3.

### Learning Integration

**Figure 5:**
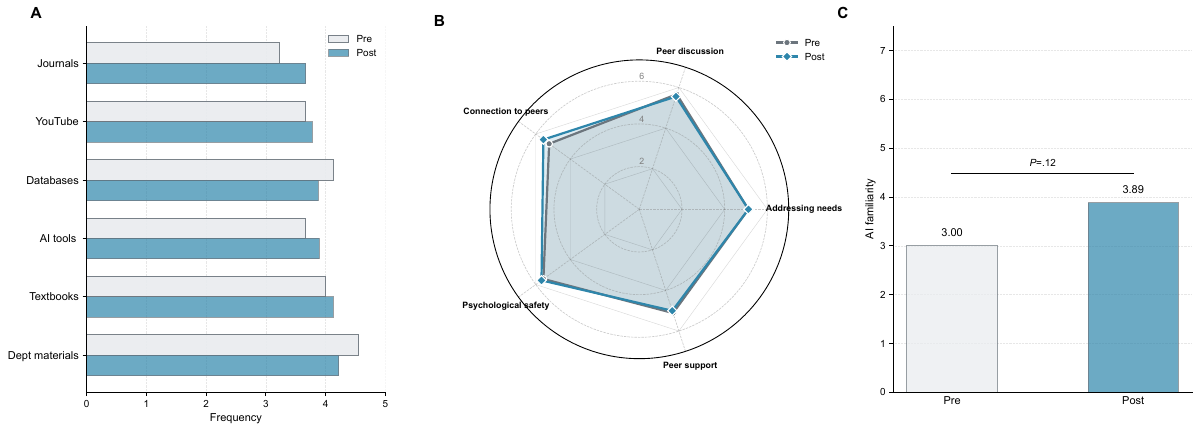
Learning context and integration outcomes. (A) Frequency of educational resource use at baseline (pre) and postintervention (post). No significant changes were observed (textbooks *P*≥.99, journal articles *P*=.25, departmental materials *P*=.25, youtube videos *P*≥.99, databases *P*=.62, and artificial intelligence tools *P*=.62). (B) Radar plot of sense-of-community dimensions, showing stable ratings across all five measures (program addressing shared needs P≥.99, peer discussion P≥.99, connection to peers *P*=.50, psychological safety *P*≥.99, and peer support *P*≥.99). (C) Self-reported artificial intelligence familiarity scores pre- and postintervention (d=0.70; *P*=.12). AI: artificial intelligence.

No significant changes in educational resource use were observed over the 12-week intervention period (textbooks *P*≥.99, journal articles *P*=.25, departmental materials *P*=.25, YouTube videos *P*≥.99, databases *P*=.62, and AI tools *P*=.62; Figure 5A; Table S5 in Multimedia Appendix 3). Weekly study time remained stable (pre median 2 hours, IQR 1-3; post median 2 hours, IQR 1.5-2; *P*=.45). Traditional resource use, including textbooks (*P*≥.99), journal articles (*P*=.25), and departmental materials (*P*=.25), did not change. This pattern suggests the intervention operated as an additive supplement rather than displacing established learning resources. All 5 sense-of-community dimensions remained stable (program addressing shared needs *P*≥.99, peer discussion *P*≥.99, connection to peers *P*=.50, psychological safety *P*≥.99, and peer support *P*≥.99; Figure 5B), with median ratings at or above 5 on the 7-point scale throughout. This stability provides preliminary evidence that asynchronous digital learning did not disrupt the apprenticeship model central to surgical training. AI tool familiarity showed the largest contextual change (pre median 3, IQR 2-5; post median 4, IQR 4-5; *d*=0.70; *P*=.12), though this did not reach statistical significance (Figure 5C).

## Discussion

### Principal Findings

This pilot study provides preliminary evidence that AI-generated microlearning modules can be feasibly integrated into surgical residency education. Over 12 weeks, all 11 enrolled residents completed postintervention assessments, and module submissions ranged from 9 to 11 across the 6 subspecialty topics. Cumulative time spent of 60 to 120 minutes was distributed across brief, self-paced sessions (10-20 minutes per module), suggesting compatibility with clinical schedules. Faculty evaluation confirmed high content quality across all modules, and postintervention acceptability ratings were uniformly positive. Among 9 matched pairs, composite self-perceived confidence improved significantly (*d*=0.65; *P*=.047), while composite knowledge showed a medium effect size (*d*=0.71) that did not reach statistical significance (*P*=.09). These preliminary findings support the feasibility of this approach and provide effect size estimates to inform the design of future confirmatory trials.

### Positioning Within the Literature

Our findings extend the emerging literature on LLM-generated medical education content. Prior studies have established that AI systems can generate MCQs achieving quality ratings comparable to human-authored items when evaluated by content experts [15,16,22]. However, most investigations have examined question quality as an endpoint rather than deploying AI-generated content within authentic educational interventions [21,22]. Our study moves beyond quality benchmarking by embedding curated AI content into a 12-week residency curriculum and measuring educational outcomes, albeit in an exploratory pilot design with a small sample.

When positioned within the broader literature, our composite effect sizes (*d*=0.65-0.71) align with the upper range of effects reported in continuing medical education interventions, where effect sizes corresponding to Cohen *d* of approximately 0.4 to 0.7 are typical [30]. These are notably smaller than effects reported in undergraduate medical education meta-analyses (SMDs of 1.2-3.4 for case-based and simulation-based learning) [31,32], likely reflecting the greater baseline knowledge and clinical experience of postgraduate learners. Against this benchmark, our preliminary findings fall within the upper range of reported continuing medical education effect sizes, despite the minimal time burden, asynchronous format, and reliance on AI-generated content.

### Predictors of Educational Effectiveness

Module-level analyses offered exploratory insight into content characteristics associated with educational outcomes. Perceived content quality (*r*=0.79) and relevance (*r*=0.69) showed strong positive associations with knowledge change, whereas perceived difficulty showed only a weak negative association (*r*=–0.31). These patterns are consistent with prior work demonstrating that perceived clinical relevance fosters knowledge retention [33]. If confirmed in larger samples, these associations carry practical implications: faculty reviewers and developers of AI-generated educational content might prioritize clinical relevance and pedagogical quality as primary selection criteria.

A consistent inverse relationship between baseline self-assessed knowledge and improvement emerged across modules (*r*=–0.85), with the largest gains in hand and peripheral nerve surgery (*d*=1.48; *P*=.02) and aesthetic and breast surgery (*d*=0.93; *P*=.06), where baseline scores were lowest. Conversely, modules with higher baseline scores showed negligible or no change (core surgical *d*=0; burns *d*=–0.23). This floor-ceiling pattern is consistent with targeted learning theory and suggests that AI-generated microlearning may be particularly suited for addressing identified knowledge gaps rather than reinforcing already-strong domains.

### Content Quality and Expert Evaluation

The structured faculty evaluation process resulted in the selection of 42 items from an initial pool of 60, elevating the mean quality index from 4.28 to 4.68 on a 5-point scale. The safety dimension remained consistently high across all modules (range 4.71-4.89) and showed negligible correlation with other quality dimensions, suggesting that LLM outputs can maintain appropriate clinical safety standards even when other aspects of content quality vary. This structural independence is noteworthy given concerns about AI-generated medical content potentially propagating harmful recommendations. Despite this independence, the expert curation process proved essential for overall content quality, suggesting that human expert oversight remains important for deploying AI-generated content in medical education contexts where content errors carry safety implications.

However, interrater reliability between the 2 faculty evaluators was poor (ICC(3,1)=0.231, 95% CI 0.154-0.305). This finding highlights the inherent subjectivity of content quality judgments and the limited precision afforded by a 2-rater design. Future implementations should consider larger evaluation panels and consensus-based approaches to improve reliability. Despite the low ICC, both raters independently selected similar items for inclusion, suggesting agreement on which items were clearly strong or clearly weak, even when absolute ratings diverged.

Internal consistency was low, with negative Cronbach ɑ values in 3 of 6 modules, reflecting the heterogeneous content within each module where items spanned distinct subtopics. The 2 modules with positive internal consistency (burns, ɑ=0.69; sarcoma, ɑ=0.74) also showed the highest item discrimination, suggesting that future item development might benefit from greater subtopic coherence within modules.

### Learning Integration

The stability of residents’ learning ecosystems throughout the intervention provides preliminary evidence that asynchronous AI-based microlearning can supplement existing educational resources without displacement. Weekly study time, traditional resource use, and all 5 sense-of-community dimensions remained unchanged, suggesting the modules functioned as an additive intervention. This nondisruption is relevant given concerns that digital learning tools might undermine the apprenticeship model central to surgical training.

AI tool familiarity showed the largest contextual change (*d*=0.70), though this did not reach statistical significance (*P*=.12). Exposure to faculty-evaluated AI content may function as experiential onboarding, potentially preparing residents for the AI-augmented clinical environments they will increasingly encounter. Whether curated AI educational content can serve as an entry point for broader AI literacy warrants investigation in future studies.

### Strengths and Limitations

This study has several methodological strengths. The rolling production model enabled continuous content delivery while maintaining rigorous faculty evaluation, demonstrating a workflow for AI-assisted curriculum development. The standardized prompting strategy and comprehensive evaluation rubric provide reproducible frameworks for other programs seeking to implement similar interventions. The multidimensional assessment captured not only efficacy outcomes but also feasibility, acceptability, and ecosystem integration metrics essential for translating pilot findings into sustainable educational practice.

Several limitations warrant consideration when interpreting these findings. First, the single-arm design without a control group precludes causal attribution; secular trends, Hawthorne effects, or concurrent clinical experiences may have contributed to observed changes. Second, the small sample size (n=9 matched pairs) limited statistical power, as reflected in wide confidence intervals, and some outcomes that showed medium-to-large effect sizes did not reach statistical significance. Third, self-reported knowledge and confidence outcomes may not reflect objective performance on standardized examinations or clinical competency assessments. Fourth, interrater reliability for the faculty evaluation was poor (ICC=0.231), limiting the precision of content quality judgments. Fifth, the single-institution setting within a German academic plastic surgery program, the 12-week follow-up period, and the reliance on a single LLM (Google Gemini 2.5 Pro) with a specific prompting strategy all constrain generalizability. However, the evaluation framework and curriculum structure remain applicable regardless of which AI system generates initial content.

### Conclusions

This pilot study provides evidence that AI-generated microlearning modules are feasible and acceptable for plastic surgery residency education. Faculty-evaluated content achieved high-quality ratings, and residents showed strong engagement with meaningful preliminary improvements in self-perceived confidence. The intervention was integrated into existing learning ecosystems without disrupting traditional resources or peer learning dynamics. The methodology described here, combining structured prompt engineering with subspecialty-specific content generation, systematic faculty evaluation, and learner-centered outcome assessment, may provide a framework for programs exploring similar AI-assisted educational interventions, though replication across institutions and specialties is needed. These findings, while preliminary, support proceeding to adequately powered randomized trials evaluating AI-generated microlearning against standard educational approaches, with attention to objective outcome measures, longer follow-up, and multi-institutional settings.

## Appendix

Multimedia Appendix 1 Artificial intelligence content generation prompt, faculty evaluation rubric with scoring criteria, and example multiple-choice questions with explanations.

Multimedia Appendix 2 Additional figures supporting the study findings.

Multimedia Appendix 3 Additional tables supporting the study findings.

## Abbreviations

AI: artificial intelligence
CONSORT: Consolidated Standards of Reporting Trials
ICC: intraclass correlation coefficient
LLM: large language model
MCQ: multiple-choice question
MWBO: Musterweiterbildungsordnung
PGY: postgraduate year

## Data Availability

All data analysis code as well as deidentified participant data are available upon reasonable request from the corresponding author. Data access may be subject to institutional review board approval.
