# Supplementary material for: AI-Generated Microlearning for Plastic Surgery Residency: Single-Arm Pre-Post Feasibility Study
Source: JMIR Med Educ. 2026 Jul 14;12:e88695. doi: 10.2196/88695 (PMC13416306; doi:10.2196/88695)
Supplement: Multimedia Appendix 1 [file mededu_v12i1e88695_app1.docx]

**Supplementary Figures for AI-Generated Microlearning for Plastic Surgery Residency: A Pilot Feasibility Study**

*Marius Drysch^1^, Sonja Verena Schmidt^1^, Felix Reinkemeier^1^, Flemming Puscz^1^, Alexander Fiedler^1^, Maria Fueth^1^, Marcus Lehnhardt^1^, Alexander Sogorski^1^, Christoph Wallner^1^*

^1^ Department of Plastic Surgery, BG University Hospital Bergmannsheil, Ruhr University Bochum, 44789 Bochum, Germany

**Corresponding Author:**

Marius Drysch, MD, MHBA

Department of Plastic Surgery

BG University Hospital Bergmannsheil

Bürkle-de-la-Camp-Platz 1

44789 Bochum, Germany

Phone: +49 234 302 0

Email: marius.drysch@rub.de

**Keywords:** Artificial Intelligence; Education, Medical, Graduate; Surgery, Plastic; Microlearning; Large Language Models; Feasibility Studies; Multiple-Choice Questions

**Supplementary Information I - AI Prompt for MCQ Generation**

# I. Persona & Core Task

You are an expert medical educator and item writer specializing in Plastic Surgery. Your primary task for this interaction is to generate one (1) unique, high-quality multiple-choice question (MCQ) and its detailed explanation for the specific Plastic Surgery subtopic that will be provided at the end of these instructions.

# II. Desired Style & Standard

The question and explanation should emulate the style, depth, clinical focus, and difficulty typically found in board review sections of a leading textbook such as "Janis Essentials of Plastic Surgery." This means the content should be:

- Direct and Board-Focused: Testing important, examinable concepts.
- Clinically Oriented: Often using concise vignettes (aim for 2-4 sentences for patient presentations where appropriate for the subtopic) that are realistic but not esoteric.
- Appropriately Challenging: Suitable for well-trained Plastic Surgery residents, assessing application of knowledge and reasoning beyond simple recall.
- High-Yield Explanations: Explanations should be clear, concise, and directly address why the correct answer is correct and why distractors are incorrect, highlighting key learning points relevant to board preparation.

# III. Critical Guidelines to Ensure

## A. Content Integrity

1. Accuracy & Currency: All medical information provided in both the question and explanation must be factually accurate, current, and reflect evidence-based guidelines and established scientific consensus in Plastic Surgery relevant to the subtopic. If uncertain about a specific detail, indicate this.
2. Patient Safety ("Do No Harm"): Absolutely no information should mislead or promote unsafe clinical practices. Omissions of critical safety warnings pertinent to the subtopic must be avoided. Reinforce principles of safe and ethical medical practice.

## B. MCQ Construction (Adhering to Best Practices)

1. Core Question Focus & Cognitive Level:

Focus on Important Concepts: The MCQ must target an important medical concept or testing point relevant to Plastic Surgery training for residents, based on the provided subtopic. Avoid trivial facts.

Assess Application of Knowledge: Primarily design MCQs that assess the application of knowledge. This often requires context through a clinical vignette. However, for subtopics centered on specific advanced anatomical structures or the detailed criteria of classification systems, direct questions testing precise recall or direct application of these structured concepts are also valuable and acceptable.

Integrate Systematic Knowledge: Each question, whether vignette-based or a direct query, must be designed so that its resolution requires the learner to engage with, recall, or apply knowledge of a specific clinical classification system (e.g., Palmer classification, Baker classification, Salter-Harris classification), pertinent detailed anatomy, or another systematic, tangible medical concept relevant to the subtopic. The scenario or question should intrinsically lead to the application or consideration of this systematic knowledge.

1. Structure: The MCQ must have exactly five (5) answer options: one unequivocally best correct answer and four plausible but incorrect distractors.
2. Clarity & Item-Writing Principles: The question stem must be clear, focused, unambiguous, and ideally "closed" (per the "cover-the-options" rule). Options should be concise, homogeneous, and parallel. Adhere to established item-writing best practices to avoid common flaws (e.g., no grammatical cues, no "always/never" in options unless truly applicable, correct answer not standing out, clang associations, etc., as detailed in NBME guidelines).
3. Cognitive Demand: Aim for Bloom's Taxonomy 'Application' level or higher (e.g., requiring analysis of complex data, synthesis of information to solve a problem, or evaluation of different approaches). Strive to create scenarios that demand multi-step reasoning, integration of knowledge from different domains, or application of detailed classification systems, diagnostic criteria, or nuanced management principles relevant to the subtopic, truly challenging a well-trained Plastic Surgery resident. The difficulty level should be appropriate for a general Plastic Surgery resident audience.

## C. Explanation Quality

1. Comprehensive Justification: Meticulously explain why the correct answer is correct AND why each of the four distractor options is incorrect, addressing potential misconceptions.
2. Highlight Systematic Knowledge: The explanation must explicitly clarify or reference the specific clinical classification, anatomical point, or systematic concept that was key to understanding and answering the question.
3. Pedagogical Elements: Link the specific subtopic to broader anatomical, physiological, or pathological principles relevant to Plastic Surgery.

## D. Ethical Considerations

1. Bias-Free Content: All content must strive for inclusive and neutral presentation.

When a medical condition within the subtopic has a clear, significant, and well-established predilection for a specific gender, the patient presented in the vignette should typically reflect that predominant gender, as this forms part of the clinically relevant presentation.

For general conditions within the subtopic without strong gender linkage, or when describing roles/scenarios not tied to a specific gender-predominant condition, vary patient characteristics (including gender) thoughtfully to reflect diverse populations and actively work to avoid perpetuating societal stereotypes.

For routine gender identification, use simple and direct language (e.g., 'a 32-year-old man,' 'a 45-year-old woman') when gender is presented as binary and is not a specific clinical focus of the vignette that requires more nuanced terminology for its core clinical point.

1. Patient Privacy: If generating a hypothetical clinical scenario for this subtopic, ensure patient privacy is maintained by using de-identified or aggregated concepts.

# IV. Required Output Format

Provide your output for the single subtopic (specified in Section VI below) clearly, structured with the following distinct section headers:

Subtopic: (Repeat the subtopic that will be provided in Section VI).

Question: (Present the full clinical vignette, if used, typically 2-4 sentences for a concise patient presentation, followed by the lead-in question. Then list the five answer options: A, B, C, D, E.)

Correct Answer: (Clearly state the letter of the correct answer for the question above. For example: "C")

Detailed Explanation: (This section must be comprehensive and clearly structured. Ideally, use sub-headings or a similar logical flow within this explanation to cover all points):

- Rationale for Correct Answer: (Provide a clear and comprehensive justification explaining why the designated correct answer is indeed correct, based on the vignette and established medical knowledge relevant to the subtopic.)
- Rationale for Incorrect Options: (Meticulously explain why each of the four distractor options is incorrect. Address potential misconceptions or reasons why a trainee might mistakenly choose them.)
- Systematic Knowledge Highlight: (Explicitly identify, define, and briefly explain the core clinical classification, pertinent detailed anatomy, diagnostic criteria, or other systematic, tangible concept that is key to understanding and answering the question, as emphasized in guideline III.B.1.)
- Broader Educational Links: (Link the specific subtopic to broader medical principles relevant to Plastic Surgery, as detailed in Section III.C.3.)

# V. Iterative Process Expectation

Your generated content will be reviewed by human experts (Plastic Surgery faculty). Be prepared for feedback based on the above criteria. Apply all principles consistently for this item.

# VI. Specific Subtopic for This Item

SUBTOPIC: [User to Insert One Specific Subtopic Here]

**Supplementary Information II - Faculty Grading Manual**

# I. Introduction & Purpose of Validation

Welcome, and thank you for lending your expertise to this pilot study on AI-generated micro-learning for Plastic Surgery residents. This study aims to evaluate the feasibility, effectiveness, and reception of using AI-generated multiple-choice questions (MCQs) and their accompanying explanations as a supplementary educational tool. The purpose of this validation process is to critically assess the quality, accuracy, clinical relevance, pedagogical value, and safety of the AI-generated content before it is presented to resident learners. Your expert judgment is crucial in ensuring that only high-quality, appropriate, and safe educational materials are utilized in this program.

# II. Overview of the AI-Generated Content to be Reviewed

You will be provided with sets of AI-generated educational items. Each item consists of:

1. A Multiple-Choice Question (MCQ): This includes a clinical scenario/stem and five answer options.
2. An Identified Correct Answer (Key).
3. A Detailed Explanation: This explains why the keyed answer is correct and why the distractor options are incorrect, aiming to provide further educational context, highlight systematic knowledge, and link to broader principles.

## Target Audience for AI Content

For this initial study, the AI has been prompted to generate questions and explanations covering core concepts and foundational knowledge relevant to all Plastic Surgery residents. While the aim is broad applicability, your feedback on the perceived suitability for different PGY levels within your qualitative comments will be valuable for future refinements.

Overarching Principle: "Do No Harm": A fundamental principle guiding your review should be "Primum Non Nocere" (First, do no harm). All content, particularly explanations, must be scrutinized for any information that could potentially mislead a trainee or promote unsafe clinical practices.

# III. The Validation Rubric: Structure & General Rating Instructions

The rubric is structured around 5 Core Dimensions of quality. Each dimension is assessed by two specific rubric items: one evaluating the AI-generated Question component and one evaluating the AI-generated Explanation component, resulting in 10 core quantitative assessments per Q&A pair. Detailed guidance, including definitions, examples of different performance levels, and specific considerations for each of these 5 dimensions and their 10 rating items, is provided in Section IV of this document to assist your evaluation.

## General Rating Instructions for Validators

Please rate each of the 10 rubric items (detailed in Section IV below) on the following 5-point Likert scale based on your initial review of the AI-generated Question and its corresponding AI-generated Explanation. Focus on evaluating the AI-generated Question and Explanation separately as indicated. The target cognitive level for the questions is Bloom's Taxonomy Application level or higher. Learning Objectives for the AI-generated content are intended to align with core Plastic Surgery training competency frameworks. Throughout your review, please ensure careful consideration of patient safety and freedom from bias.

## Rating Scale

- 5 = Excellent: The item meets all expectations for this criterion to a high degree; no significant concerns.
- 4 = Good: The item meets expectations well; any issues are minor and easily addressable.
- 3 = Average: The item is largely acceptable but has some clear aspects that could or should be improved.
- 2 = Fair: The item meets expectations minimally; significant revisions are required to make it acceptable.
- 1 = Poor: The item does not meet expectations for this criterion; major revisions are needed, or the item may need to be rejected.

## Qualitative Comments

(Optional but highly encouraged): Following each numerical rating for the 10 rubric items, you have the option to provide specific, actionable qualitative comments. While optional, these comments are highly encouraged, particularly for ratings of 3 (Average) or lower, or when you have specific suggestions for improvement. Your narrative feedback is important for:

- Explaining the reasoning behind your scores.
- Identifying specific strengths and weaknesses of the AI-generated content.
- Offering concrete suggestions for revision.
- Noting any nuanced observations (e.g., "While factually accurate, this concept might be too advanced for very junior residents without further context," or "Distractor B is problematic because...").

# IV. Detailed Guidance for Each Rubric Dimension & Item

## Dimension 1: Accuracy & Currency

This dimension fundamentally asks: Is the content correct and up-to-date? It assesses the factual correctness and timeliness of the medical information.

### 1.1. Rubric Item (for Question)

"How accurate and current is the medical content of the Question?"

Detailed Guidance for Validators: Evaluate all components of the AI-generated Question – the clinical scenario/stem, all answer options (A, B, C, D, E), and the identified correct answer (key) – for factual correctness. Assess if this information aligns with current, broadly accepted scientific consensus and evidence-based Plastic Surgery practice. Is the terminology precise and current? Identify any factual errors, outdated information, or misleading statements within the question itself.

Excellent (5) Example: All parts of the question are factually indisputable, terminology is precise and current, and the keyed answer is definitively correct based on current evidence.

Poor (1) Example: The question stem contains significant factual errors, uses outdated terminology that changes meaning, or the keyed correct answer is incorrect/debatable.

### 1.2. Rubric Item (for Explanation)

"How accurate and current is the medical content of the Explanation?"

Detailed Guidance for Validators: Scrutinize the entire AI-generated Explanation for factual accuracy and consistency with current medical understanding and evidence-based guidelines. Pay attention to details such as pathophysiology, anatomical descriptions, diagnostic criteria, treatment modalities, and prognoses mentioned. Is the information up-to-date, especially in rapidly evolving areas of Plastic Surgery?

Excellent (5) Example: The explanation is entirely factually sound, up-to-date, references concepts correctly, and provides information consistent with current best practices.

Poor (1) Example: The explanation contains significant factual errors, promotes outdated or disproven concepts, or misrepresents medical data/evidence.

## Dimension 2: Relevance & Objective Alignment

This dimension fundamentally asks: Is the content important and targeted for our learners? It focuses on the pertinence of the content to Plastic Surgery training (for a general Plastic Surgery resident audience) and its alignment with stated learning outcomes.

### 2.1. Rubric Item (for Question)

"How clinically relevant is the Question's topic/scope to Plastic Surgery training and how well does it align with standard Learning Objectives?"

Detailed Guidance for Validators: Consider if the topic tested by the AI-generated Question is important ("must-know" or "important-to-know") for a competent general Plastic Surgery resident. Does it reflect common or critical clinical problems, foundational knowledge, or essential skills outlined in core Plastic Surgery training competency frameworks (e.g., EBOPRAS)? Assess how directly the question targets the provided learning objective(s).

Excellent (5) Example: The question addresses a highly significant clinical concept or core principle directly aligned with standard learning objectives and is clearly relevant for a general Plastic Surgery resident.

Poor (1) Example: The question tests an obscure or trivial topic with limited relevance to general Plastic Surgery training, or it significantly deviates from standard learning objectives.

### 2.2. Rubric Item (for Explanation)

"How well does the Explanation support the Learning Objective(s) and enhance understanding of clinical relevance?"

Detailed Guidance for Validators: Evaluate if the AI-generated Explanation not only clarifies the answer but also reinforces why the topic is clinically important for a Plastic Surgery resident and how it helps achieve the stated learning objective(s). Does it help the learner connect the concept to practical aspects of patient care, decision-making, or core competencies?

Excellent (5) Example: The explanation clearly links the item's content back to the learning objective(s) and effectively illustrates its clinical significance.

Poor (1) Example: The explanation is purely factual recall without connecting to the learning objective(s) or highlighting any clinical context or relevance.

## Dimension 3: Clarity & Quality of Construction

This dimension fundamentally asks: Is the content well-written and understandable in its structure? It focuses on the linguistic quality and structural soundness of the question and the explanation.

### 3.1. Rubric Item (for Question)

"How clear, precise, and well-constructed is the Question (stem, options, distractors)?"

Detailed Guidance for Validators: Assess the AI-generated Question for linguistic clarity, freedom from ambiguity, grammatical correctness, and appropriate terminology. The stem should present a focused problem. Options should be parallel in structure where possible. Critically evaluate distractors: Are they plausible yet unequivocally incorrect? Do they represent common errors or misconceptions? Are they free of common item-writing flaws (e.g., 'giveaways,' significant length discrepancies, technical jargon differences, convergence, absurd options that don't test knowledge)? Ensure there is only one best answer.

Excellent (5) Example: The question is impeccably worded, stem is focused, options are clear and parallel, distractors are high quality (plausible but clearly wrong), and there is one undeniable best answer. Free of item-writing flaws.

Poor (1) Example: The question stem is vague or confusing. Options are awkwardly phrased or ambiguous. Distractors are non-functional or flawed. Multiple correct answers possible, or no clear best answer.

### 3.2. Rubric Item (for Explanation)

"How clear, organized, and well-written is the Explanation in language and structure?"

Detailed Guidance for Validators: Evaluate the AI-generated Explanation for its linguistic quality. Is it written in clear, concise, and professional language suitable for medical residents? Is it well-organized with a logical flow of information? Is it free of grammatical errors, spelling mistakes, or awkward phrasing that could impede understanding?

Excellent (5) Example: The explanation is exceptionally clear, logically structured, well-written, and easy for a resident to understand.

Poor (1) Example: The explanation is disorganized, convoluted, contains numerous grammatical errors, or uses language that is confusing or inappropriately informal.

## Dimension 4: Pedagogical Value

This dimension fundamentally asks: Does it effectively teach and promote deeper learning? It assesses the educational effectiveness of the item in promoting thought, assessing appropriate skills, managing difficulty, and ensuring the explanation is comprehensive and deepens understanding.

### 4.1. Rubric Item (for Question)

"What is the pedagogical value of the Question in stimulating thought, assessing appropriate cognitive skills (Application level or higher), and its difficulty level?"

Detailed Guidance for Validators: Consider if the AI-generated Question promotes learning beyond simple recall. Does it require application of knowledge, analysis, or clinical reasoning (targeting Bloom's Taxonomy 'Application' level or higher for core concepts)? Is the difficulty level appropriate for a general Plastic Surgery resident audience (i.e., challenging yet achievable)? Does the question itself make the learner think critically?

Excellent (5) Example: The question effectively assesses higher-order thinking or critical application of core knowledge at an appropriate level of challenge for a general Plastic Surgery resident and stimulates thoughtful consideration.

Poor (1) Example: The question tests trivial recall, is far too easy or impossibly difficult for the target audience, or does not effectively assess meaningful understanding or application at an appropriate cognitive level.

### 4.2. Rubric Item (for Explanation)

"What is the pedagogical value of the Explanation in comprehensively justifying answers, offering educational depth, and promoting further learning/critical thinking?"

Detailed Guidance for Validators: Assess if the AI-generated Explanation is comprehensive in covering why the correct answer is right and, importantly, why each distractor is wrong, addressing potential misconceptions. Does it provide sufficient educational depth by linking to underlying principles or relevant clinical context? Does it include elements (e.g., brief discussion of related concepts, implications) that might encourage residents to think more deeply?

Excellent (5) Example: The explanation is thorough, insightful, clearly justifies all options, connects concepts effectively, and promotes deeper understanding.

Poor (1) Example: The explanation is superficial, merely re-states the answer, fails to adequately explain distractors, lacks depth, and does not promote intellectual engagement.

## Dimension 5: Ethical & Safety Considerations

This dimension fundamentally asks: Is it fair and safe to use? It addresses the fairness of the content (freedom from bias) and its safety (upholding the "do no harm" principle, with explicit attention to patient safety).

### 5.1. Rubric Item (for Question)

"Is the Question free from demographic, cultural, or other biases, and does it avoid any misleading content affecting patient safety?"

Detailed Guidance for Validators: Scrutinize the AI-generated Question (stem, scenario, options) for any language or assumptions that could reflect or perpetuate bias related to age, gender, race/ethnicity, socioeconomic status, culture, etc. Ensure it is inclusive. Crucially, assess if any aspect of the question could inadvertently lead to a misunderstanding that compromises patient safety or sound clinical reasoning.

Excellent (5) Example: The question is entirely neutral, fair, inclusive, and presents no risk regarding patient safety understanding.

Poor (1) Example: The question contains biased language/stereotypes or presents a scenario in a way that could inadvertently lead to a clinically unsafe interpretation or conclusion.

### 5.2. Rubric Item (for Explanation)

"Is the Explanation free from bias and does it avoid misleading or unsafe clinical recommendations affecting patient safety?"

Detailed Guidance for Validators: Similar to the question, evaluate the AI-generated Explanation for any bias. Most importantly, assess if the explanation contains any information that is misleading from a safety perspective, omits critical warnings or contraindications pertinent to patient safety, or could otherwise promote clinical understanding or practices that are not aligned with optimal patient safety.

Excellent (5) Example: The explanation is neutral, fair, and all information presented is consistent with safe and ethical clinical practice; critical patient safety considerations are appropriately handled.

Poor (1) Example: The explanation contains biased information, or provides advice/information that is clinically unsafe, omits crucial safety details, or could be easily misinterpreted to lead to patient harm.

# V. Calibration Process & Achieving Consistency

To ensure that all validators are applying these criteria in a consistent manner, we will undertake a calibration process:

1. Group Review of this Document: We will begin with a group meeting (virtual or in-person) to discuss this Briefing Document and the Validation Rubric in detail, addressing any questions or ambiguities.
2. Independent Rating of Sample Items: A common set of 3-5 sample AI-generated question-explanation pairs (not part of the actual study set) will be provided. Each validator will independently rate these items using the rubric.
3. Facilitated Discussion & Consensus Building: We will then reconvene to discuss the ratings for these sample items. This discussion will focus on identifying discrepancies in ratings, understanding the reasoning behind different scores, clarifying interpretations of the rubric criteria and anchors, and working towards a shared understanding and a consensus approach for applying the rubric.
4. Ongoing Communication: Throughout the validation process, please feel free to reach out if you encounter any ambiguous items or have questions about applying the rubric.

# VI. Practicalities of the Validation Task

- You will receive the AI-generated question-explanation pairs and the link to the online rating form in batches of 7 questions.
- It is recommended that you have the document containing the Q&A pairs and the online scoring sheet open simultaneously to facilitate efficient review.

# VII. Concluding Remarks

Careful review and feedback are essential for the success of our project and for ensuring the quality and safety of the AI-generated learning materials. Let's make sure that they are as good and safe as they should be.

**Supplementary Information III – Examples of MCQs**

# 1. Hand Surgery

## Question 1: Metacarpal Fractures

A 30-year-old man presents to the emergency department after falling onto his outstretched hand with his thumb abducted. He complains of significant pain, swelling, and bruising localized to the base of his right thumb. Radiographs reveal an oblique intra-articular fracture of the base of the first metacarpal. A small volar-ulnar fragment remains aligned with the trapezium, while the larger distal metacarpal shaft fragment is displaced radially, dorsally, and proximally.

**Which of the following muscles is primarily responsible for the characteristic displacement of the major first metacarpal fragment in this fracture pattern?**

A) Extensor pollicis longus (EPL)

B) Adductor pollicis

C) Flexor pollicis longus (FPL)

D) First dorsal interosseous

E) Abductor pollicis longus (APL)

**Correct Answer: E**

### Detailed Explanation

**Rationale for Correct Answer:** Abductor pollicis longus (APL): The fracture pattern described is a classic Bennett's fracture, which is an intra-articular fracture-dislocation of the base of the first metacarpal. The Abductor pollicis longus (APL) tendon inserts onto the radial aspect of the base of the first metacarpal. In a Bennett's fracture, the strong volar oblique ligament (part of the ulnar collateral ligament complex, sometimes referred to as the "beak ligament") holds the small volar-ulnar fragment in its anatomical position relative to the trapezium. The unopposed pull of the APL on the larger, unstable metacarpal shaft fragment causes it to displace radially, dorsally, and proximally.

**Rationale for Incorrect Options:**

- Extensor pollicis longus (EPL): The EPL primarily extends the interphalangeal joint of the thumb and contributes to extension and adduction at the metacarpophalangeal (MP) and carpometacarpal (CMC) joints. While it influences thumb position, it is not the primary deforming force causing the specific proximal, dorsal, and radial displacement of the main fragment in a Bennett's fracture.
- Adductor pollicis: The Adductor pollicis is a powerful muscle that adducts the thumb towards the palm and has insertions on the ulnar sesamoid, proximal phalanx, and often the ulnar aspect of the first metacarpal base/shaft. Its pull can contribute to adduction and supination deformity of the first metacarpal shaft and can exacerbate instability, particularly in more comminuted fractures like a Rolando fracture, or contribute to the difficulty in maintaining reduction. However, the primary force causing the classic dorsal, radial, and proximal migration of the main metacarpal shaft in a Bennett's fracture is the APL.
- Flexor pollicis longus (FPL): The FPL flexes the interphalangeal and metacarpophalangeal joints of the thumb. Its line of pull would tend to flex the metacarpal, not cause the characteristic dorsal and proximal displacement seen.
- First dorsal interosseous: This muscle originates from the adjacent sides of the first and second metacarpals and inserts on the radial base of the proximal phalanx of the index finger. It has no direct pull on the first metacarpal shaft that would cause the described displacement.

**Systematic Knowledge Highlight:** This question tests knowledge of the Bennett's fracture, a specific type of intra-articular fracture of the base of the first metacarpal, and the anatomical forces that cause its characteristic displacement. Bennett's Fracture Definition: An unstable, oblique intra-articular fracture of the base of the first metacarpal, where the shaft displaces due to muscle forces while a small volar-ulnar fragment (the "Bennett's fragment") is held in place by the strong anterior oblique ligament (volar beak ligament) attached to the trapezium. Key Anatomical Structures & Deforming Forces: The Anterior Oblique Ligament (Volar Beak Ligament) stabilizes the volar-ulnar fragment to the trapezium. The Abductor Pollicis Longus (APL) inserts on the radial base of the first metacarpal, and its unopposed pull on the shaft fragment causes displacement dorsally, radially, and proximally. The Adductor Pollicis can contribute to adduction and supination of the distal fragment, further complicating reduction and stability.

**Broader Educational Links:** This subtopic connects to broader principles in hand surgery: Intra-articular Fractures require accurate anatomic reduction to minimize the risk of post-traumatic arthritis, pain, and stiffness, especially in a critical joint like the thumb CMC joint. The thumb CMC joint is a saddle joint crucial for opposition, grasp, and pinch. Instability or arthrosis here can severely impair hand function. Understanding the pull of various extrinsic and intrinsic muscles is essential for diagnosing fracture displacement patterns and planning treatment to counteract deforming forces. Bennett's fractures are inherently unstable due to the fracture pattern and muscle forces, often requiring surgical fixation (e.g., closed reduction and percutaneous pinning, or open reduction and internal fixation) to achieve and maintain an anatomical reduction.

## Question 2: Ganglion Cyst

A 38-year-old female administrative assistant presents with a 4-month history of a gradually enlarging, non-tender, 1.5 cm mass on the dorsum of her left wrist. The mass is firm and mobile. She reports occasional mild aching after prolonged typing but denies any specific trauma. Physical examination reveals no overlying skin changes or neurovascular compromise. An ultrasound confirms a simple cystic structure without internal vascularity.

**Which of the following structures is the most common specific point of origin for this patient's presentation?**

A) Scapholunate interosseous ligament

B) Radioscaphocapitate ligament

C) Extensor pollicis longus tendon sheath

D) Lunotriquetral interosseous ligament

E) Dorsal radiocarpal ligament

**Correct Answer: A**

### Detailed Explanation

**Rationale for Correct Answer:** Scapholunate interosseous ligament: This is the correct answer. The most common type of ganglion cyst is the dorsal wrist ganglion, accounting for approximately 60-70% of all wrist and hand ganglions. These cysts characteristically arise from the dorsal aspect of the scapholunate interosseous ligament and the adjacent dorsal radiocarpal joint capsule. The clinical presentation of a non-tender, firm mass on the dorsal wrist, confirmed as cystic by ultrasound, is classic for this entity.

**Rationale for Incorrect Options:**

- Radioscaphocapitate ligament: The radioscaphocapitate ligament is a key volar stabilizer of the wrist. While volar wrist ganglia (the second most common type) can arise from volar ligaments and joint capsules (often near the radial artery and related to the radioscaphocapitate or scaphotrapezial joints), the patient's presentation is clearly a dorsal wrist ganglion.
- Extensor pollicis longus (EPL) tendon sheath: While tenosynovitis or ganglia can arise from tendon sheaths, a ganglion originating specifically from the EPL tendon sheath is not the most common origin for a dorsal wrist mass as described. Furthermore, EPL pathology often presents with pain or dysfunction related to thumb movement, which is not described. Dorsal wrist ganglia are typically associated with the underlying carpal joints and ligaments.
- Lunotriquetral interosseous ligament: Ganglions can arise from the lunotriquetral (LT) interosseous ligament, but this is a less common origin for dorsal wrist ganglia compared to the scapholunate interosseous ligament. LT-originating ganglia are often located slightly more ulnarly on the dorsal wrist.
- Dorsal radiocarpal ligament: The dorsal radiocarpal ligament is a broad structure covering the dorsal aspect of the radiocarpal joint. While a dorsal ganglion is intimately related to this ligament and the underlying joint capsule, the most common specific focal point of origin is more precisely attributed to the scapholunate interosseous ligament region. The question asks for the most common specific point of origin, making the scapholunate ligament a more precise answer for the classic dorsal wrist ganglion.

**Systematic Knowledge Highlight:** Common Locations and Origins of Wrist Ganglions: Dorsal Wrist Ganglion (60-70%) most commonly arises from the scapholunate interosseous ligament and the dorsal joint capsule. Volar Wrist Ganglion (15-20%) typically arises from the radiocarpal joint, scaphotrapezial joint, or surrounding volar ligaments, often located adjacent to the radial artery. Flexor Tendon Sheath Ganglion (Retinacular Cyst) presents as small, firm, often tender nodules typically found at the base of a finger over the A1 pulley region. Mucous Cyst (DIP Joint Ganglion) occurs at the dorsal aspect of the DIP joint, often associated with underlying osteoarthritis. Pathophysiology: Ganglion cysts are not true cysts as they lack an epithelial lining. They are pseudocysts filled with viscous, mucinous fluid. The prevailing theory is that they result from mucinous degeneration of periarticular connective tissue with a one-way valve-like mechanism.

**Broader Educational Links:** It's important to differentiate ganglion cysts from other soft tissue masses of the wrist and hand, such as lipomas, giant cell tumors of the tendon sheath, vascular malformations, or (rarely) malignant tumors. While transillumination is a classic physical exam finding, imaging such as ultrasound is highly effective in confirming the cystic nature of the mass. Management options range from observation to aspiration (with a high recurrence rate) to surgical excision. Surgical excision involves tracing the ganglion stalk to its point of origin and excising a small portion of the adjacent capsule or ligament to reduce recurrence. Understanding the precise anatomical origins is crucial for effective surgical planning.

# 2. Skin Grafts & Microsurgery

## Question 3: STSG vs. FTSG

A 68-year-old woman undergoes Mohs micrographic surgery for a basal cell carcinoma, resulting in a 1.5 cm circular defect of the left lower eyelid tarsal plate and overlying skin. The surgeon is planning reconstruction with a skin graft.

**Which of the following statements most accurately describes the expected clinical behavior of the ideal graft choice for this location?**

A) A split-thickness graft is preferred and will exhibit high primary contraction and low secondary contraction.

B) A full-thickness graft is preferred and will exhibit high primary contraction and low secondary contraction.

C) A split-thickness graft is preferred due to its more rapid revascularization, minimizing the risk of graft failure on the eyelid.

D) A full-thickness graft is preferred and will exhibit low primary contraction and high secondary contraction.

E) Either graft type is acceptable, as secondary contraction is primarily dependent on the recipient wound bed rather than the graft type.

**Correct Answer: B**

### Detailed Explanation

**Rationale for Correct Answer:** A full-thickness graft is preferred and will exhibit high primary contraction and low secondary contraction. For reconstructive defects on the eyelid, a full-thickness skin graft (FTSG) is the superior choice. The eyelid is a mobile, cosmetically sensitive area where functional outcomes are paramount. The primary advantage of an FTSG in this location is its minimal secondary contraction, which significantly reduces the risk of postoperative ectropion (eversion of the eyelid margin), a serious functional and aesthetic complication. An FTSG includes the entire epidermis and dermis. High primary contraction occurs due to the high content of dermal elastin fibers upon harvesting. Low secondary contraction occurs because the FTSG brings its own thick dermal scaffold, making it more resistant to the contractile forces of myofibroblasts.

**Rationale for Incorrect Options:**

- A split-thickness skin graft (STSG) is not preferred for the eyelid due to its high rate of secondary contraction. Furthermore, its contraction properties are the opposite of what is stated; an STSG exhibits low primary contraction and high secondary contraction.
- While it is true that an STSG generally revascularizes more quickly and reliably than an FTSG (due to its thinness), it is not the preferred choice here. The severe functional deficit (ectropion) caused by its high secondary contraction outweighs the marginal benefit of more rapid "take." The face and eyelids have an excellent blood supply, making successful FTSG take highly probable.
- This option correctly identifies the preferred graft type (FTSG) but incorrectly describes its behavior. An FTSG has high primary and low secondary contraction. This option describes the behavior of an STSG.
- This statement is incorrect and represents a significant misconception. The type of graft—specifically its dermal thickness—is a critical determinant of the degree of secondary contraction. An STSG, containing little dermis, provides a poor barrier to underlying myofibroblast-mediated wound contraction.

**Systematic Knowledge Highlight:** This question hinges on the critical concepts of Primary vs. Secondary Skin Graft Contraction, which are directly related to the anatomical composition of the graft. Skin Graft Composition: STSG contains the full epidermis and a variable portion of the dermis; FTSG contains the full epidermis and the entire dermis. Primary Contraction is the immediate, passive elastic recoil upon harvesting, directly proportional to dermal thickness (FTSG = high, STSG = low). Secondary Contraction is the active, long-term size reduction over weeks and months, inversely proportional to dermal thickness (STSG = high up to 50%, FTSG = low).

**Broader Educational Links:** This topic directly links to fundamental principles of wound healing and tissue biology. The process of secondary contraction is a manifestation of normal wound healing, driven by the differentiation of fibroblasts into myofibroblasts. The choice between an STSG and FTSG requires the surgeon to balance the properties of the graft (contraction, durability, pigmentation) with the needs of the recipient site (vascularity, cosmetic importance, functional demands) and the morbidity of the donor site. Understanding these trade-offs is fundamental to reconstructive plastic surgery.

## Question 4: Medicinal Leeches

A 28-year-old man undergoes successful replantation of an avulsed right index finger. The arterial repair is robust, but due to severe vessel damage, only a single, small-caliber dorsal vein could be anastomosed. Twelve hours post-operatively, the replanted digit is warm but exhibits severe violaceous discoloration and tense swelling, consistent with critical venous congestion. Medicinal leech therapy is initiated. While the volume of blood consumed by the leech itself is small, the primary therapeutic benefit is derived from the sustained, prolonged local bleeding that occurs after the leech detaches.

**This effect is chiefly mediated by which component of leech saliva?**

A) Hyaluronidase

B) A potent anesthetic agent

C) Calin and collagenase

D) Histamine-like vasodilators

E) Hirudin

**Correct Answer: E**

### Detailed Explanation

**Rationale for Correct Answer:** Hirudin: The primary mechanism by which leeches provide sustained relief from venous congestion is the profound and localized anticoagulation they induce. The most important bioactive compound responsible for this effect is hirudin, a highly specific and potent direct inhibitor of thrombin. Thrombin is a critical enzyme in the final common pathway of the coagulation cascade, responsible for converting fibrinogen to fibrin to form a stable clot. By inhibiting thrombin, hirudin prevents the formation of a clot at the bite site, allowing the wound to ooze venous blood for up to 8-12 hours after the leech has detached. This prolonged micro-phlebotomy (often 20-50 mL per bite site) is the cornerstone of the therapy, effectively decompressing the congested tissue and allowing for the development of alternative venous outflow channels.

**Rationale for Incorrect Options:**

- Hyaluronidase is a crucial enzyme in leech saliva, but it is not the primary anticoagulant. Its role is that of a "spreading factor." It breaks down hyaluronic acid in the extracellular matrix, increasing local tissue permeability. This allows the other active components of the saliva, including hirudin, to diffuse more widely and effectively into the surrounding tissues. Its action is synergistic, but it is not the principal agent of sustained bleeding.
- Leech saliva does contain anesthetic compounds, which is why their bite is often painless. This facilitates feeding for the leech by preventing the host from detecting it. However, anesthesia has no role in promoting blood flow or preventing coagulation and is not the source of the therapeutic effect in relieving congestion.
- While these substances are present and contribute to the overall effect, they are not the chief mediators. Calin is a platelet adhesion inhibitor that works by preventing von Willebrand factor from binding to collagen. Collagenase can break down collagen. Both actions interfere with the initial stages of platelet plug formation, but hirudin's direct inhibition of the fibrin clot formation is the dominant and most powerful mechanism for sustained anticoagulation.
- Leech saliva contains histamine-like substances that cause local vasodilation. This helps increase blood flow to the bite site, making feeding easier for the leech. While this contributes, the vasodilation itself does not prevent the blood from clotting. The prolonged bleeding is an issue of failed hemostasis, not just increased vessel diameter.

**Systematic Knowledge Highlight:** This question requires a systematic understanding of the key bioactive molecules in the saliva of the medicinal leech, Hirudo medicinalis. The key components include: Hirudin (the most potent natural direct thrombin inhibitor, responsible for sustained anticoagulation); Hyaluronidase (a "spreading factor" increasing tissue permeability); Calin (an inhibitor of collagen-induced platelet aggregation); Apyrase (a platelet aggregation inhibitor); Destabilase (an enzyme with some fibrinolytic properties); and Anesthetics & Vasodilators (making the bite painless and increasing local blood flow). The core testing point is to differentiate between the primary actor (hirudin) and these important but secondary/synergistic agents.

**Broader Educational Links:** This topic connects to fundamental principles of microsurgery, physiology, and pharmacology. It highlights a classic challenge in digital replantation, where venous outflow is often more difficult to establish and more prone to failure than arterial inflow. Answering correctly requires knowledge of the hemostasis pathway, specifically recognizing thrombin's central role in converting fibrinogen to a fibrin clot. It provides a biological example of targeted drug action, contrasting the direct thrombin inhibition of hirudin with other anticoagulant mechanisms.

# 3. Burn Surgery

## Question 5: Pathophysiology of Burn Shock

A 34-year-old man sustains 50% TBSA flame burns and arrives at the emergency department within one hour of the injury. Fluid resuscitation has just been initiated. A pulmonary artery catheter is placed for hemodynamic monitoring.

**Which set of hemodynamic measurements is most characteristic of this initial post-burn period?**

A) High Cardiac Index, Low Systemic Vascular Resistance, High Pulmonary Capillary Wedge Pressure

B) Low Cardiac Index, High Systemic Vascular Resistance, Low Pulmonary Capillary Wedge Pressure

C) Low Cardiac Index, Low Systemic Vascular Resistance, High Pulmonary Capillary Wedge Pressure

D) High Cardiac Index, High Systemic Vascular Resistance, Normal Pulmonary Capillary Wedge Pressure

E) Normal Cardiac Index, Low Systemic Vascular Resistance, Normal Pulmonary Capillary Wedge Pressure

**Correct Answer: B**

### Detailed Explanation

**Rationale for Correct Answer:** Low Cardiac Index (CI), High Systemic Vascular Resistance (SVR), Low Pulmonary Capillary Wedge Pressure (PCWP): This is the classic hemodynamic profile of the initial "ebb phase" (the first 24-48 hours) of burn shock. The question describes the patient "within one hour of injury," which places them squarely in this phase. The massive thermal injury triggers the release of inflammatory mediators that cause a dramatic increase in systemic capillary permeability, leading to a rapid loss of protein-rich plasma fluid from the intravascular space into the interstitium. Low PCWP reflects decreased venous return due to this profound fluid loss. Low CI results from decreased preload (per the Frank-Starling mechanism) and myocardial depressant factors like TNF-α. High SVR is the body's compensatory response through massive sympathetic nervous system discharge, causing intense peripheral vasoconstriction to maintain vital organ perfusion.

**Rationale for Incorrect Options:**

- High CI, Low SVR, High PCWP describes a hyperdynamic, distributive shock state, most characteristic of the later "flow phase" of burn injury (after successful resuscitation) or septic shock. It is the opposite of the initial presentation.
- Low CI, Low SVR, High PCWP is characteristic of decompensated cardiogenic shock. The high PCWP indicates volume overload or primary pump failure, and the low SVR suggests a loss of vascular tone, neither of which is the primary driver of the initial phase of burn shock.
- High CI, High SVR, Normal PCWP is a physiologically inconsistent profile. It is nearly impossible to have a high cardiac output against a high systemic vascular resistance.
- Normal CI, Low SVR, Normal PCWP may represent a mild, early distributive shock but fails to capture the profound hypovolemia (low PCWP) and compensatory vasoconstriction (high SVR) that define severe, unresuscitated burn shock.

**Systematic Knowledge Highlight:** The Biphasic Cardiovascular Response to Burns: The systemic response to a major burn follows a distinct two-phase pattern. The Ebb Phase (Initial 24-48 hours) is the period of burn shock, characterized by hypovolemia, hypometabolism, and vasoconstriction. Key features include decreased cardiac output, increased vascular resistance, and reduced oxygen consumption. The Flow Phase (begins after resuscitation, can last for weeks/months) is the period of hypermetabolism, characterized by a hyperdynamic, hypermetabolic, and catabolic state. Key features include high cardiac output, low systemic vascular resistance, increased heart rate, and massively increased energy expenditure and protein breakdown.

**Broader Educational Links:** The pathophysiology of burn shock is a direct illustration of the disruption of Starling's forces governing fluid exchange across capillaries. The burn injury damages the capillary endothelium, drastically increasing its permeability (the filtration coefficient). This allows both water and large protein molecules like albumin to leak into the interstitium, raising interstitial oncotic pressure and diminishing plasma oncotic pressure. This explains why simple crystalloid resuscitation (like the Parkland formula) is required in such large volumes—it is needed to replace the lost intravascular volume until the capillary integrity is gradually restored.

## Question 6: Pathogens in Burn Injury

A 32-year-old woman is hospitalized after sustaining 30% TBSA deep partial-thickness scald burns to her legs and abdomen. She is managed with topical silver sulfadiazine and daily dressing changes. On post-burn day 7, she develops a fever of 39.1°C (102.4°F) and tachycardia. During wound care, the nurse notes that the dressings over her legs have a faint bluish-green discoloration and a distinct, sweet, "grape-like" odor. The underlying wound bed appears soupy, and previously adherent portions of the eschar are now liquefying and separating.

**What is the most likely causative organism?**

A) Staphylococcus aureus

B) Pseudomonas aeruginosa

C) Enterococcus faecalis

D) Aspergillus fumigatus

E) Klebsiella pneumoniae

**Correct Answer: B**

### Detailed Explanation

**Rationale for Correct Answer:** Pseudomonas aeruginosa is the quintessential opportunistic gram-negative pathogen in burn wounds, classically emerging during the intermediate phase of colonization (5-14 days post-burn). The clinical picture described is pathognomonic for a Pseudomonas burn wound infection. The bluish-green discoloration is caused by its production of water-soluble pigments, primarily pyocyanin (blue-green) and pyoverdin (yellow-green and fluorescent). The characteristic sweet, "grape-like" or "tortilla chip" odor is also highly suggestive. Furthermore, Pseudomonas produces potent exotoxins and proteases that lead to tissue liquefaction, vascular invasion (ecthyma gangrenosum), and rapid graft loss, consistent with the "soupy" appearance and eschar separation described.

**Rationale for Incorrect Options:**

- Staphylococcus aureus is a gram-positive organism that typically colonizes the wound earlier. Infections usually present with thick, creamy, odorless pus and surrounding cellulitis rather than pigmented, watery discharge.
- Enterococcus faecalis, a gram-positive coccus originating from the gut, can colonize burn wounds, especially in critically ill patients. However, it does not produce pigments or a characteristic odor and is not typically associated with the rapid liquefactive necrosis described.
- Aspergillus fumigatus is a mold that causes invasive infection in the late phase of burn care (typically >2 weeks), often in severely immunocompromised patients. It presents as a focal, dry, black necrotic patch due to angioinvasion, which is a starkly different presentation.
- Klebsiella pneumoniae is another important gram-negative rod that can cause burn wound infections. It typically produces copious, mucoid, and sometimes blood-tinged exudate but does not create the classic green pigment or fruity odor associated with Pseudomonas.

**Systematic Knowledge Highlight:** This question tests the critical concept of the temporal succession of pathogens in burn wounds. The timing of the infection on post-burn day 7 is a key clue. The burn wound environment evolves from being dominated by gram-positive skin flora (Staphylococcus) in the first few days to being overtaken by gram-negative bacteria (Pseudomonas, Klebsiella, E. coli) from the patient's own gut flora or the hospital environment. The vignette is a classic example of an infection arising in this intermediate, gram-negative-dominant period.

**Broader Educational Links:** This scenario underscores the importance of microbial virulence factors in clinical diagnosis. The pigments of Pseudomonas (pyocyanin, pyoverdin) are not merely diagnostic curiosities; they are virulence factors that induce oxidative stress and impair host cell function. This pathogen's destructive capability is also linked to its secretion of enzymes like elastase and alkaline protease, and toxins like Exotoxin A (which inhibits protein synthesis similarly to diphtheria toxin), explaining the rapid tissue and graft destruction. This highlights why burn wound care emphasizes not only systemic antibiotics but also aggressive topical control to suppress the microbial bioburden at the wound surface.

# 4. Aesthetic & Breast Surgery

## Question 7: Classification of Breast Ptosis

A 38-year-old woman presents for evaluation for a mastopexy. On physical examination with the patient standing, her nipple is located 2 cm below the inframammary fold, but it is above the most dependent portion of the breast parenchyma.

**According to the Regnault classification of breast ptosis, what is her grade of ptosis?**

A) Grade I

B) Grade II

C) Grade III

D) Pseudoptosis

E) Parenchymal maldistribution

**Correct Answer: B**

### Detailed Explanation

**Rationale for Correct Answer:** Grade II ptosis is defined as the nipple being located below the inframammary fold (IMF), but still above the lower contour of the breast. The Regnault classification is based on the relationship between the nipple position and the inframammary fold.

**Rationale for Incorrect Options:**

- Grade I ptosis (mild) is defined by the nipple being at the level of the IMF, but above the lower breast contour.
- Grade III ptosis (severe) is defined by the nipple being below the IMF and at the most dependent point of the breast, pointing downwards.
- Pseudoptosis is characterized by the nipple being at or above the IMF, but the majority of the breast parenchyma hangs below the fold, giving the appearance of sagging. This patient's nipple is below the IMF.
- Parenchymal maldistribution is a descriptive term, not a formal grade in the Regnault classification. It often accompanies ptosis but does not define its grade.

**Systematic Knowledge Highlight:** The Regnault Classification of Breast Ptosis is a foundational system for standardizing the description of breast sagging and guiding surgical planning. The grades are: Pseudoptosis (Nipple above IMF, breast tissue sags below); Grade I/Mild (Nipple at IMF); Grade II/Moderate (Nipple below IMF, but above the most dependent breast tissue); Grade III/Severe (Nipple below IMF and at the lowest point of the breast).

**Broader Educational Links:** Correctly classifying ptosis is essential for selecting the appropriate surgical technique. Pseudoptosis or Grade I ptosis may be correctable with augmentation alone. Grade II and III ptosis almost always require a formal mastopexy (e.g., vertical, inverted-T) to reposition the NAC and excise excess skin.

## Question 8: Seroma Formation After Abdominoplasty

A 50-year-old patient develops a large, persistent seroma following a traditional abdominoplasty with wide undermining.

**Which of the following is the most effective and widely adopted surgical maneuver to prevent seroma formation during the initial operation?**

A) Use of closed-suction drains for at least three weeks

B) Application of a compression binder for six weeks

C) Meticulous electrocautery of all lymphatic channels

D) Placement of progressive tension sutures (PTS)

E) Prescribing bed rest for the first postoperative week

**Correct Answer: D**

### Detailed Explanation

**Rationale for Correct Answer:** A seroma is a collection of serous fluid that forms in the dead space between the abdominal flap and the rectus fascia. The most effective technique to prevent this is to obliterate the dead space. Progressive tension sutures (PTS), as described by Pollock, achieve this by incrementally suturing the underside of the abdominal flap down to the underlying muscle fascia. This quilting process closes the dead space, reduces shearing forces, and distributes tension away from the final incision line. Studies have shown that PTS significantly reduces seroma rates, often to the point where drains are no longer necessary.

**Rationale for Incorrect Options:**

- Drains can be used to treat a dead space but they are less effective in preventing seroma formation than PTS.
- Compression binders help reduce swelling but cannot reliably obliterate a large dead space on their own.
- It is impossible to identify and cauterize all lymphatic channels.
- Bed rest is not practical and increases the risk of VTE without reliably preventing seroma.

**Systematic Knowledge Highlight:** Progressive Tension Sutures (PTS) / Quilting Sutures: This technique has become a standard of care in abdominoplasty. By closing the dead space, it provides several benefits: dramatically reduces seroma and hematoma rates; may eliminate the need for surgical drains; and distributes tension across the flap, potentially improving blood flow and reducing tension on the final skin closure, leading to better scars.

**Broader Educational Links:** The principle of dead space management is critical across all fields of surgery. Any time a large potential space is created (e.g., after mastectomy, latissimus flap harvest, or extensive soft tissue debridement), the surgeon must have a plan to manage it. Options include drains, compression dressings, and tissue apposition techniques like quilting sutures. Obliterating dead space is key to preventing fluid collections and subsequent infections.

# 5. Sarcoma & Oncologic Surgery

## Question 9: Radiotherapy-Induced Changes

A patient is planned for delayed reconstruction of a chest wall defect six months after completing a course of radiation therapy for a sarcoma. During dissection, the surgeon notes that the tissue planes are indistinct, fibrotic, and bleed easily with minor manipulation.

**This is a direct result of which late effect of radiation?**

A) Acute inflammatory cell infiltration

B) Endarteritis obliterans and tissue fibrosis

C) Increased tissue elasticity

D) Hyperplasia of fibroblast progenitor cells

E) Temporary reduction in tissue perfusion

**Correct Answer: B**

### Detailed Explanation

**Rationale for Correct Answer:** The classic late changes of radiation on tissue are progressive fibrosis and vascular damage in the form of endarteritis obliterans. The radiation damages the endothelial cells of small blood vessels, leading to narrowing and eventual occlusion of their lumens. This chronic ischemia, combined with direct radiation-induced fibroblast dysfunction, results in the deposition of dense, disorganized collagen. This creates the characteristic clinical findings of stiff, woody, fibrotic tissue planes that are difficult to dissect and have poor intrinsic healing capacity.

**Rationale for Incorrect Options:**

- Acute inflammatory infiltration is an early effect of radiation, seen in the days to weeks following treatment, not a late effect seen at six months.
- Radiation causes a profound loss of tissue elasticity due to fibrosis, not an increase.
- Radiation depletes fibroblast populations and impairs their function, leading to hypocellularity, not hyperplasia.
- The reduction in tissue perfusion caused by endarteritis obliterans is permanent and progressive, not temporary.

**Systematic Knowledge Highlight:** The effects of radiation on tissue are bimodal: acute and late. Acute effects (during and shortly after RT) are due to damage to rapidly dividing cells (e.g., skin epidermis, mucosa) and are characterized by inflammation, erythema, and desquamation. These are generally reversible. Late effects (months to years after RT) are due to damage to slowly dividing stromal and vascular cells. They are characterized by fibrosis, vascular obliteration (endarteritis), and tissue atrophy. These changes are progressive and irreversible.

**Broader Educational Links:** Understanding radiation-induced tissue injury is critical in any surgical field that interfaces with oncology. The same process of endarteritis and fibrosis is responsible for osteoradionecrosis (ORN) of the jaw, radiation cystitis of the bladder, and radiation proctitis of the rectum. The treatment for severe ORN is resection of the necrotic bone and reconstruction with a vascularized bone flap (e.g., fibula), reinforcing the principle that the only way to reverse the effects of radiation is to replace the damaged tissue with healthy, well-vascularized tissue from outside the radiation field.

## Question 10: Postpartum Abdominal Wall Mass

A 31-year-old woman, 6 months postpartum from her second pregnancy, presents for evaluation of a firm, non-tender mass in her right lower abdomen. It seems to be located within the abdominal wall musculature. She first noticed it about a month ago and thinks it is getting larger. She denies any fever, weight loss, or skin changes. On physical exam, there is a 6-cm, firm, immobile mass palpable deep to the skin, just lateral to the edge of her rectus abdominis muscle. Tensing the abdominal wall makes the mass more prominent and fixed.

**This clinical history and finding are most characteristic of which of the following?**

A) Hematoma

B) Lipoma

C) Leiomyosarcoma

D) Incisional hernia

E) Desmoid tumor (Aggressive Fibromatosis)

**Correct Answer: E**

### Detailed Explanation

**Rationale for Correct Answer:** This is the classic presentation of an abdominal wall desmoid tumor. The key differentiators are: 1) Demographics: A woman of childbearing age. 2) History: Strong association with pregnancy (postpartum) and prior abdominal surgery, both of which involve trauma/stretching of the fascia. 3) Location: Arising from the musculoaponeurotic structures of the abdominal wall. 4) Palpation: A rock-hard, infiltrative mass. While benign (they do not metastasize), they are locally very aggressive and have a high rate of recurrence.

**Rationale for Incorrect Options:**

- A hematoma would typically follow a specific traumatic event, would be tender, and would be expected to resolve or organize over time, not grow.
- A lipoma is a soft, mobile, "doughy" tumor of fat that is not fixed to the muscle.
- A leiomyosarcoma of the abdominal wall is very rare and would not have this strong association with pregnancy.
- An incisional hernia would present as a reducible bulge with a palpable fascial defect, not a solid mass.

**Systematic Knowledge Highlight:** The Pathogenesis of Desmoid Fibromatosis: The core concept is that desmoids are fibroblastic neoplasms linked to abnormalities in the Wnt/APC signaling pathway. They are associated with: Sporadic cases (often have mutations in the CTNNB1 gene/beta-catenin); Familial Adenomatous Polyposis (FAP)/Gardner Syndrome (caused by germline mutations in the APC gene); and Trauma and High Estrogen States (pregnancy is a classic trigger). The clinical utility is in risk factor recognition. A new abdominal wall mass in a postpartum woman or a patient with a history of FAP should immediately raise high suspicion for a desmoid, prompting imaging (MRI is best) and core needle biopsy for diagnosis.

**Broader Educational Links:** This case illustrates the principle of hormonal influence on tumor growth. While not a classic endocrine organ, the fibroblasts in a desmoid tumor often express estrogen receptors, and their growth can be stimulated by the high-estrogen state of pregnancy. This concept is most famously applied in breast cancer, where estrogen receptor (ER) positive tumors are treated with anti-estrogen therapies like tamoxifen or aromatase inhibitors. It is also seen in the growth of uterine fibroids (leiomyomas).

# 6. Patient Safety & Perioperative Care

## Question 11: Venous Thromboembolism

A 48-year-old female with a BMI of 32 kg/m² underwent an abdominoplasty with muscle plication lasting 4.5 hours. Her Caprini score was calculated to be 5, and she was placed on enoxaparin 40 mg daily and sequential compression devices (SCDs). On postoperative day 3, she develops acute-onset shortness of breath, pleuritic chest pain, and tachycardia to 110 bpm. Her oxygen saturation is 92% on room air.

**What is the most appropriate next step in management?**

A) Increase enoxaparin to a therapeutic dose of 1 mg/kg twice daily.

B) Administer a fluid bolus and obtain a lower extremity venous duplex ultrasound.

C) Obtain a stat computed tomography angiogram (CTA) of the chest.

D) Order a D-dimer test to rule out pulmonary embolism (PE).

E) Discharge the patient with reassurance as these are common anxiety symptoms.

**Correct Answer: C**

### Detailed Explanation

**Rationale for Correct Answer:** This patient's presentation is highly suspicious for an acute pulmonary embolism (PE), a life-threatening postoperative complication. Given her hemodynamic stability but clear signs of respiratory compromise, the definitive diagnostic test is a CTA of the chest. This will rapidly confirm or exclude the diagnosis and allow for prompt initiation of appropriate therapy.

**Rationale for Incorrect Options:**

- While therapeutic anticoagulation is the treatment for a confirmed PE, it should not be initiated empirically without a confirmed diagnosis, especially in a recent postoperative patient with bleeding risks. Diagnosis must come first.
- A venous duplex is used to diagnose DVT, and while the source of a PE is often a DVT, up to 30% of patients with PE have negative duplex scans. The primary life-threat is in the chest, so imaging the chest is the priority.
- The D-dimer test has high sensitivity but low specificity. It is useful for ruling out VTE in low-risk patients. In a high-risk postoperative patient with classic symptoms, the pre-test probability is high, and a positive D-dimer is expected and non-diagnostic. Proceeding directly to definitive imaging is the standard of care.
- Dismissing these classic signs of PE as anxiety would be a critical medical error with potentially fatal consequences.

**Systematic Knowledge Highlight:** The core concept is the diagnostic algorithm for suspected pulmonary embolism. This is guided by the patient's pre-test probability and hemodynamic stability. For a hemodynamically stable patient with high clinical suspicion (like this postoperative patient), the algorithm is straightforward: proceed directly to CTA of the chest. If the patient were unstable (hypotensive), the algorithm would shift to bedside echocardiography to look for right heart strain, followed by consideration of thrombolysis or embolectomy.

**Broader Educational Links:** This question connects to the foundational physiological principle of ventilation/perfusion (V/Q) mismatch. A pulmonary embolism is a classic cause of V/Q mismatch. A large clot obstructs blood flow to a segment of the lung, creating an area that is ventilated but not perfused (dead space ventilation). The blood is shunted to other perfused areas, but the overall gas exchange efficiency of the lung plummets, leading to hypoxemia. Another clinical example of V/Q mismatch is seen in acute asthma exacerbation, where bronchoconstriction prevents air from reaching the alveoli, creating a shunt (an area that is perfused but not ventilated).

## Question 12: Tranexamic Acid Mechanism

A 28-year-old woman is undergoing a cosmetic abdominoplasty. Following induction of general anesthesia, she receives 1 gram of intravenous tranexamic acid (TXA).

**What is the primary mechanism of action by which TXA reduces surgical bleeding?**

A) It directly activates platelets, promoting primary plug formation.

B) It inhibits plasminogen activation, thus preventing fibrinolysis.

C) It potentiates the effects of thrombin on fibrinogen.

D) It acts as a cofactor for Vitamin K-dependent clotting factors.

E) It causes direct vasoconstriction of small arterioles.

**Correct Answer: B**

### Detailed Explanation

**Rationale for Correct Answer:** Tranexamic acid (TXA) is a synthetic lysine analogue. Its primary mechanism of action is to act as an antifibrinolytic. It competitively inhibits the activation of plasminogen to plasmin by binding to the lysine binding sites on plasminogen. Plasmin is the key enzyme responsible for degrading fibrin clots (fibrinolysis). By preventing the breakdown of newly formed clots at the surgical site, TXA helps maintain hemostasis and reduces blood loss.

**Rationale for Incorrect Options:**

- TXA does not have a direct effect on platelet activation. Platelet function is the domain of antiplatelet drugs (like aspirin) and agonists.
- TXA does not interact with or potentiate thrombin.
- TXA is not involved in the Vitamin K-dependent coagulation pathway.
- TXA is not a vasoconstrictor. Epinephrine is the classic vasoconstrictor used in local anesthetic solutions.

**Systematic Knowledge Highlight:** The core concept is the pharmacology of hemostatic agents. These can be broadly categorized: 1) Antifibrinolytics (TXA, aminocaproic acid) which stabilize clots; 2) Procoagulants (recombinant Factor VIIa, PCC) which promote clot formation; 3) Topical agents (oxidized cellulose, gelatin sponge, topical thrombin) which provide a scaffold or direct stimulus for clotting at the bleeding surface. The clinical utility of TXA has been proven in numerous surgical fields (cardiac, orthopedic, trauma, and plastic surgery) to reduce blood loss, decrease transfusion requirements, and in some cases, lower the risk of hematoma.

**Broader Educational Links:** This question relates to the foundational physiological principle of hemostasis, which involves a balance between clot formation (coagulation) and clot breakdown (fibrinolysis). While coagulation is essential to stop bleeding, fibrinolysis is equally important to dissolve clots once they are no longer needed. A pathological state can result from a defect in either system (e.g., hemophilia from a coagulation defect; DIC can involve overwhelming fibrinolysis). TXA works by therapeutically shifting this balance away from fibrinolysis and toward clot preservation. A different example of manipulating this balance is in the treatment of acute ischemic stroke, where tissue plasminogen activator (tPA) is administered to promote rapid fibrinolysis and dissolve the thrombus.
