# Supplementary material for: AI-Generated Microlearning for Plastic Surgery Residency: Single-Arm Pre-Post Feasibility Study
Source: JMIR Med Educ. 2026 Jul 14;12:e88695. doi: 10.2196/88695 (PMC13416306; doi:10.2196/88695)
Supplement: Multimedia Appendix 2 [file mededu_v12i1e88695_app2.docx]

# **Supplementary Figures for AI-Generated Microlearning for Plastic Surgery Residency: A Pilot Feasibility Study**

*Marius Drysch^1^, Sonja Verena Schmidt^1^, Felix Reinkemeier^1^, Flemming Puscz^1^, Alexander Fiedler^1^, Maria Fueth^1^, Marcus Lehnhardt^1^, Alexander Sogorski^1^, Christoph Wallner^1^*

^1^ Department of Plastic Surgery, BG University Hospital Bergmannsheil, Ruhr University Bochum, 44789 Bochum, Germany

**Corresponding Author:**

Marius Drysch, MD, MHBA

Department of Plastic Surgery

BG University Hospital Bergmannsheil

Bürkle-de-la-Camp-Platz 1

44789 Bochum, Germany

Phone: +49 234 302 0

Email: marius.drysch@rub.de

**Keywords:** Artificial Intelligence; Education, Medical, Graduate; Surgery, Plastic; Microlearning; Large Language Models; Feasibility Studies; Multiple-Choice Questions

**
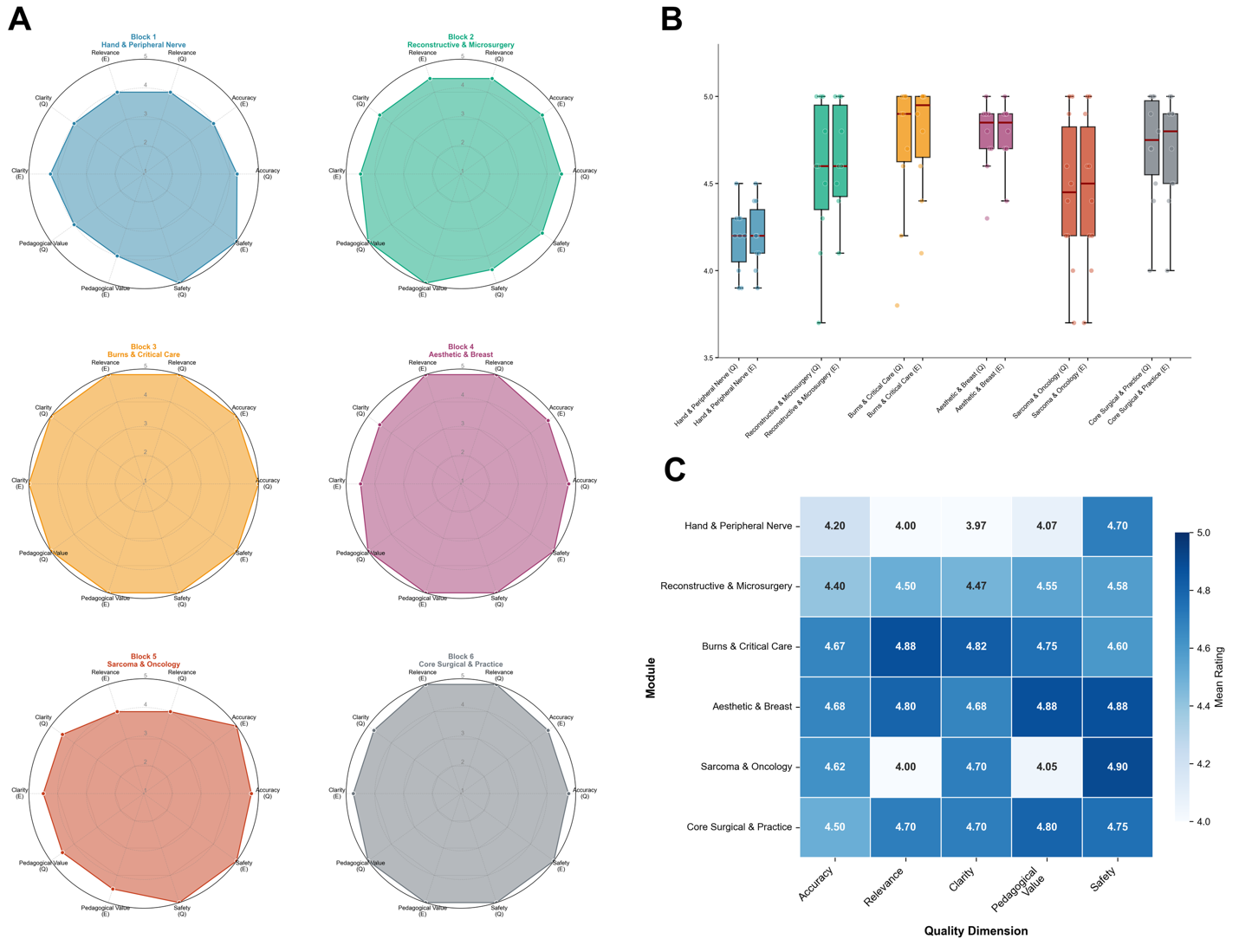
**

**Supplementary Figure 1.** Expert validation of all generated questions (n=60). (A) Radar charts displaying mean quality ratings (scale 1–5) across five dimensions (Accuracy, Relevance, Clarity, Pedagogical Value, Safety) for the final 42 selected items. Separate traces indicate ratings for generated Questions (Q) and their corresponding Explanations (E) within each of the six subspecialty modules. (B) Box plots showing the distribution of Quality Index scores for Questions (Q) and Explanations (E). Center lines represent medians, box limits indicate the interquartile range, and whiskers represent the range. The overlapping distributions highlight the close alignment between the quality of the question stems and the AI-generated explanations. (C) Heatmap of mean ratings for each quality dimension across modules. Darker blue shades indicate higher ratings.

**
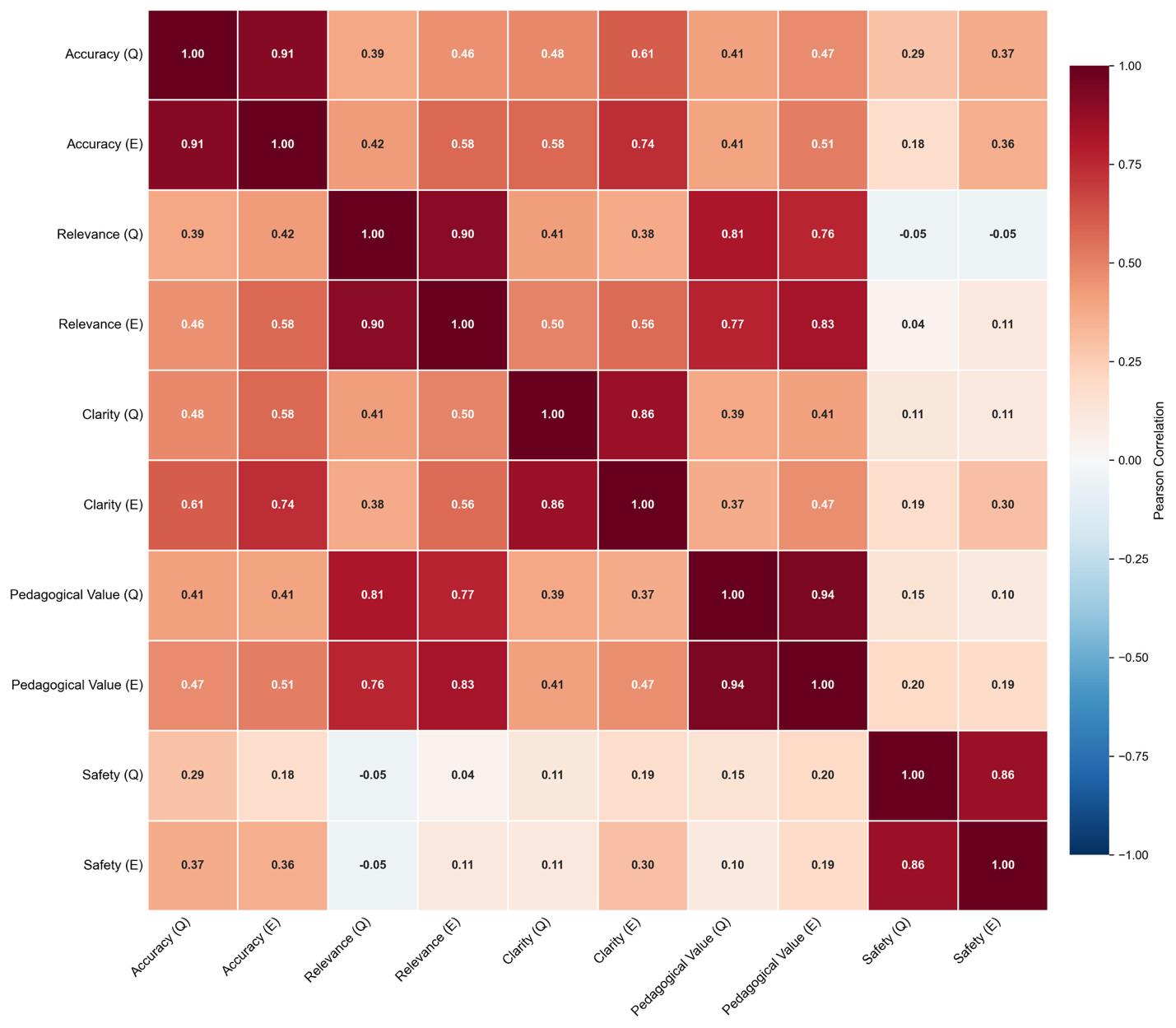
**

**Supplementary Figure 2.** Correlation matrix of quality assessment domains for complete dataset (n=60). Heatmap displaying Pearson correlation coefficients (r) among the five evaluated metrics: Accuracy, Relevance, Clarity, Pedagogical Value, and Safety. Ratings are presented separately for the generated Questions (Q) and Explanations (E). The numerical values and color gradient represent the strength and direction of the correlation, ranging from dark red (strong positive correlation, r=1.00) to dark blue (strong negative correlation).


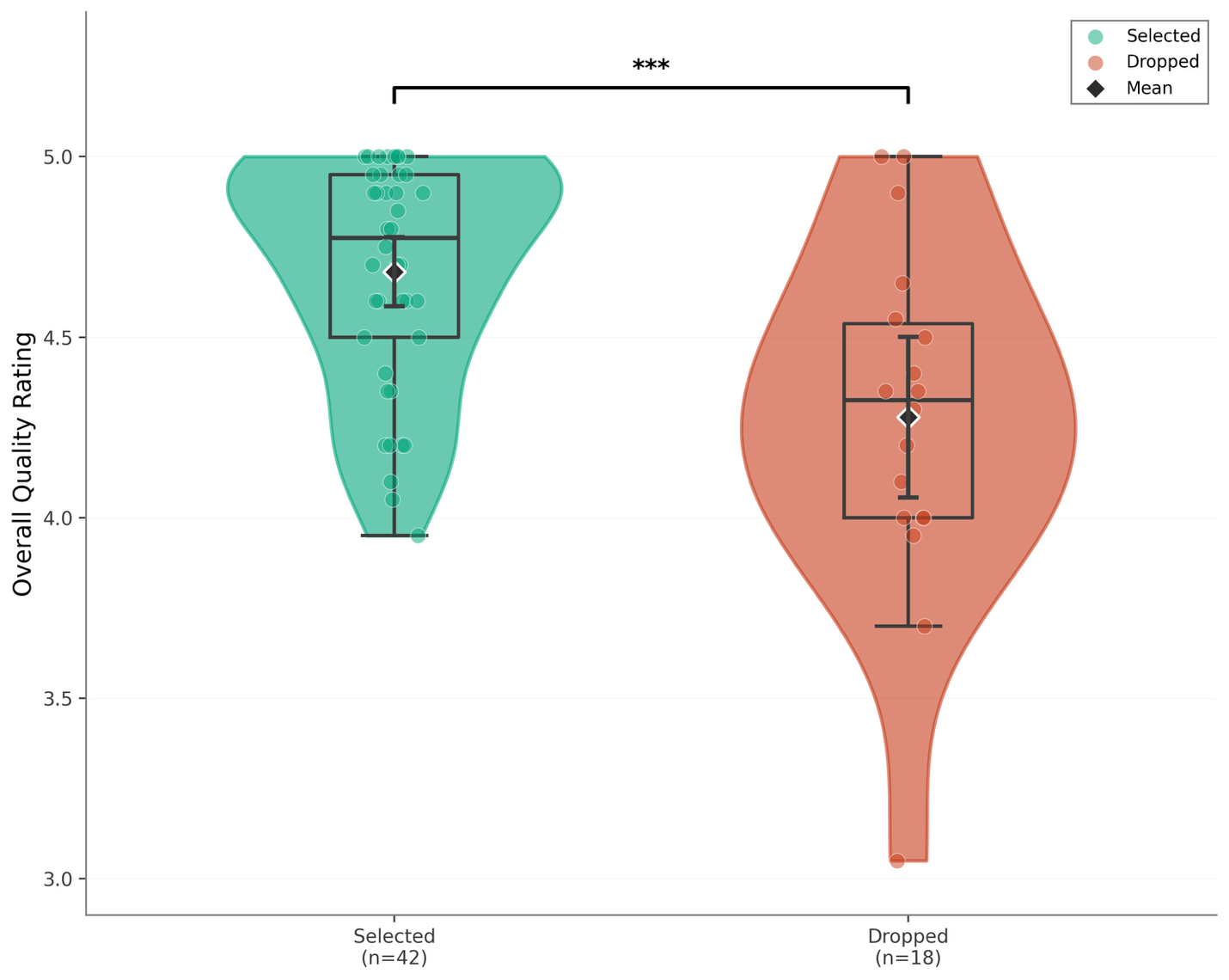


**Supplementary Figure S3.** Impact of faculty validation on quality score distributions. Violin plots comparing the mean quality ratings of the questions selected for the final pilot (n=42) versus those excluded during faculty review (n=18). The visualization displays the probability density (violin shape), median and interquartile range (internal box plot), and individual data points. Black diamonds indicate the mean score for each cohort. The selected cohort demonstrates significantly higher mean quality and a tighter distribution compared to the excluded group, which exhibits wider variance and lower scores (***p<0.001).

###
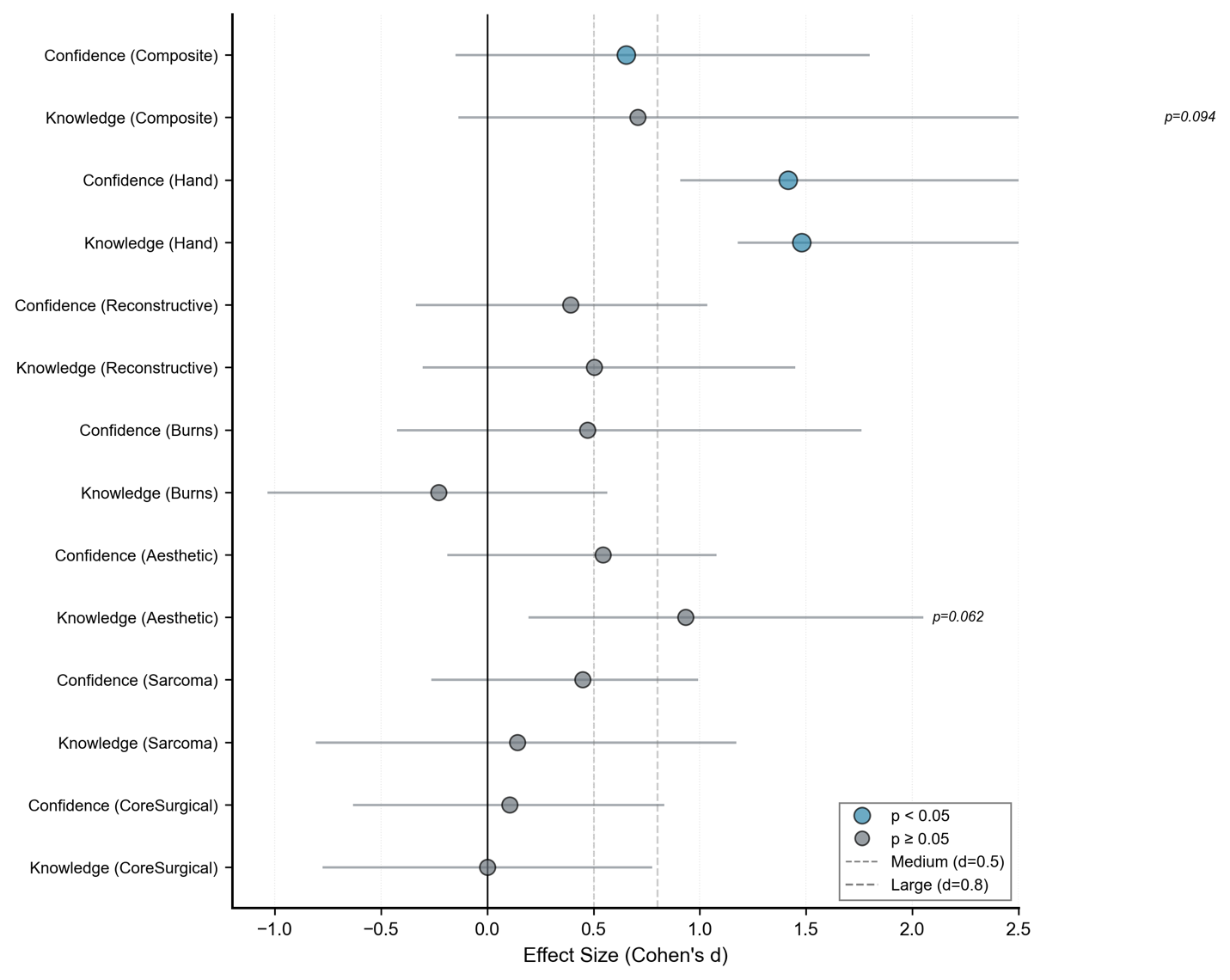


### **Supplementary Figure S4.** Forest plot ranking effect sizes across all knowledge and confidence domains. Forest plot illustrating Cohen d effect sizes representing the magnitude of change from pre- to post-intervention for composite scores and individual subspecialty modules among matched participants (n=9). Circles indicate the point estimate for Cohen d, and horizontal bars represent the 95% CIs. Markers are color-coded based on statistical significance: blue indicates significant improvement (P<0.05), and grey indicates nonsignificant change (P≥0.05). The specific p-values for the composite measures, which trended toward significance, are labelled directly on the plot. Vertical dashed lines act as reference markers for conventional interpretations of medium (d=0.5) and large (d=0.8) effect sizes.
