# Supplementary material for: AI-Generated Microlearning for Plastic Surgery Residency: Single-Arm Pre-Post Feasibility Study
Source: JMIR Med Educ. 2026 Jul 14;12:e88695. doi: 10.2196/88695 (PMC13416306; doi:10.2196/88695)
Supplement: Multimedia Appendix 3 [file mededu_v12i1e88695_app3.docx]

# Supplementary Tables for AI-Generated Microlearning for Plastic Surgery Residency: A Pilot Feasibility Study

*Marius Drysch^1^, Sonja Verena Schmidt^1^, Felix Reinkemeier^1^, Flemming Puscz^1^, Alexander Fiedler^1^, Maria Fueth^1^, Marcus Lehnhardt^1^, Alexander Sogorski^1^, Christoph Wallner^1^*

^1^ Department of Plastic Surgery, BG University Hospital Bergmannsheil, Ruhr University Bochum, 44789 Bochum, Germany

**Corresponding Author:**

Marius Drysch, MD, MHBA

Department of Plastic Surgery

BG University Hospital Bergmannsheil

Bürkle-de-la-Camp-Platz 1

44789 Bochum, Germany

Phone: +49 234 302 0

Email: marius.drysch@rub,de

**Keywords:** Artificial Intelligence; Education, Medical, Graduate; Surgery, Plastic; Microlearning; Large Language Models; Feasibility Studies; Multiple-Choice Questions

**Supplementary Table S1. Post-intervention acceptability ratings and content engagement.**

| **Item** | **Question** | **Response Option** | **Count** | **Percent** | **n** | **Mean** | **SD** | **Median** | **Min** | **Max** |
| --- | --- | --- | --- | --- | --- | --- | --- | --- | --- | --- |
| Content Engagement | How carefully did you engage with the module content? |  |  |  | 11.0 | 3.09 | 0.54 | 3.0 | 2.0 | 4.0 |
|  |  | I answered the questions quickly | 0.0 | 0.0% |  |  |  |  |  |  |
|  |  | I answered the questions without deep reflection | 1.0 | 9.1% |  |  |  |  |  |  |
|  |  | I answered the questions and reflected on the explanations | 8.0 | 72.7% |  |  |  |  |  |  |
|  |  | I answered, reflected, and sometimes looked up related topics | 2.0 | 18.2% |  |  |  |  |  |  |
|  |  | I answered, reflected deeply, and frequently consulted additional sources | 0.0 | 0.0% |  |  |  |  |  |  |
| Time per Module | On average, how much time did you spend on each module? |  |  |  | 11.0 |  |  |  |  |  |
|  |  | <10 minutes | 2.0 | 18.2% |  |  |  |  |  |  |
|  |  | 10–20 minutes | 7.0 | 63.6% |  |  |  |  |  |  |
|  |  | 20–40 minutes | 1.0 | 9.1% |  |  |  |  |  |  |
|  |  | 40–60 minutes | 1.0 | 9.1% |  |  |  |  |  |  |
|  |  | >60 minutes | 0.0 | 0.0% |  |  |  |  |  |  |
| Overall Value | This program was a valuable addition to my residency education. |  |  |  | 11.0 | 5.27 | 1.1 | 5.0 | 3.0 | 7.0 |
|  |  | Strongly Disagree | 0.0 | 0.0% |  |  |  |  |  |  |
|  |  | Disagree | 0.0 | 0.0% |  |  |  |  |  |  |
|  |  | Somewhat Disagree | 1.0 | 9.1% |  |  |  |  |  |  |
|  |  | Neutral | 1.0 | 9.1% |  |  |  |  |  |  |
|  |  | Somewhat Agree | 4.0 | 36.4% |  |  |  |  |  |  |
|  |  | Agree | 4.0 | 36.4% |  |  |  |  |  |  |
|  |  | Strongly Agree | 1.0 | 9.1% |  |  |  |  |  |  |
| Knowledge Gaps | The AI-generated MCQs helped me effectively identify my knowledge gaps. |  |  |  | 11.0 | 5.36 | 0.92 | 5.0 | 4.0 | 7.0 |
|  |  | Strongly Disagree | 0.0 | 0.0% |  |  |  |  |  |  |
|  |  | Disagree | 0.0 | 0.0% |  |  |  |  |  |  |
|  |  | Somewhat Disagree | 0.0 | 0.0% |  |  |  |  |  |  |
|  |  | Neutral | 2.0 | 18.2% |  |  |  |  |  |  |
|  |  | Somewhat Agree | 4.0 | 36.4% |  |  |  |  |  |  |
|  |  | Agree | 4.0 | 36.4% |  |  |  |  |  |  |
|  |  | Strongly Agree | 1.0 | 9.1% |  |  |  |  |  |  |
| Knowledge Improvement | Participating in this pilot project has improved my knowledge base in the topics covered. |  |  |  | 11.0 | 5.0 | 1.18 | 5.0 | 3.0 | 7.0 |
|  |  | Strongly Disagree | 0.0 | 0.0% |  |  |  |  |  |  |
|  |  | Disagree | 0.0 | 0.0% |  |  |  |  |  |  |
|  |  | Somewhat Disagree | 1.0 | 9.1% |  |  |  |  |  |  |
|  |  | Neutral | 3.0 | 27.3% |  |  |  |  |  |  |
|  |  | Somewhat Agree | 3.0 | 27.3% |  |  |  |  |  |  |
|  |  | Agree | 3.0 | 27.3% |  |  |  |  |  |  |
|  |  | Strongly Agree | 1.0 | 9.1% |  |  |  |  |  |  |
| Engagement | The micro-learning format was an engaging way to study complex topics. |  |  |  | 11.0 | 5.27 | 1.19 | 6.0 | 3.0 | 7.0 |
|  |  | Strongly Disagree | 0.0 | 0.0% |  |  |  |  |  |  |
|  |  | Disagree | 0.0 | 0.0% |  |  |  |  |  |  |
|  |  | Somewhat Disagree | 1.0 | 9.1% |  |  |  |  |  |  |
|  |  | Neutral | 2.0 | 18.2% |  |  |  |  |  |  |
|  |  | Somewhat Agree | 2.0 | 18.2% |  |  |  |  |  |  |
|  |  | Agree | 5.0 | 45.5% |  |  |  |  |  |  |
|  |  | Strongly Agree | 1.0 | 9.1% |  |  |  |  |  |  |
| Usability | The program's format was user-friendly and easy to navigate. |  |  |  | 11.0 | 5.91 | 0.83 | 6.0 | 4.0 | 7.0 |
|  |  | Strongly Disagree | 0.0 | 0.0% |  |  |  |  |  |  |
|  |  | Disagree | 0.0 | 0.0% |  |  |  |  |  |  |
|  |  | Somewhat Disagree | 0.0 | 0.0% |  |  |  |  |  |  |
|  |  | Neutral | 1.0 | 9.1% |  |  |  |  |  |  |
|  |  | Somewhat Agree | 1.0 | 9.1% |  |  |  |  |  |  |
|  |  | Agree | 7.0 | 63.6% |  |  |  |  |  |  |
|  |  | Strongly Agree | 2.0 | 18.2% |  |  |  |  |  |  |
| Continuation Intent | If this program were to continue, would you like to participate again? |  |  |  | 11.0 | 5.64 | 1.63 | 6.0 | 2.0 | 7.0 |
|  |  | Definitely not | 0.0 | 0.0% |  |  |  |  |  |  |
|  |  | Very unlikely | 1.0 | 9.1% |  |  |  |  |  |  |
|  |  | Unlikely | 0.0 | 0.0% |  |  |  |  |  |  |
|  |  | Neutral | 1.0 | 9.1% |  |  |  |  |  |  |
|  |  | Likely | 3.0 | 27.3% |  |  |  |  |  |  |
|  |  | Very likely | 1.0 | 9.1% |  |  |  |  |  |  |
|  |  | Definitely yes | 5.0 | 45.5% |  |  |  |  |  |  |

*Acceptability items rated on a 7-point Likert scale (1 = strongly disagree to 7 = strongly agree). Content engagement rated on a 5-point ordinal scale. Continuation intent rated on a 7-point scale (1 = definitely not to 7 = definitely yes).*

**Supplementary Table S2. Item-level faculty evaluation ratings for all 60 AI-generated questions.**

| **Module** | **Question ID** | **Question Text** | **Rater1 Accuracy Q** | **Rater1 Accuracy E** | **Rater1 Relevance Q** | **Rater1 Relevance E** | **Rater1 Clarity Q** | **Rater1 Clarity E** | **Rater1 Pedagogical Q** | **Rater1 Pedagogical E** | **Rater1 Safety Q** | **Rater1 Safety E** | **Rater2 Accuracy Q** | **Rater2 Accuracy E** | **Rater2 Relevance Q** | **Rater2 Relevance E** | **Rater2 Clarity Q** | **Rater2 Clarity E** | **Rater2 Pedagogical Q** | **Rater2 Pedagogical E** | **Rater2 Safety Q** | **Rater2 Safety E** | **Mean Overall** |
| --- | --- | --- | --- | --- | --- | --- | --- | --- | --- | --- | --- | --- | --- | --- | --- | --- | --- | --- | --- | --- | --- | --- | --- |
| M1: Hand & Peripheral Nerve | M1_Q1 | Metacarpal Fractures | 4.0 | 4.0 | 3.0 | 3.0 | 4.0 | 4.0 | 3.0 | 3.0 | 5.0 | 5.0 | 5.0 | 4.0 | 4.0 | 4.0 | 4.0 | 5.0 | 5.0 | 5.0 | 5.0 | 5.0 | 4.2 |
| M1: Hand & Peripheral Nerve | M1_Q2 | Nerve reconstruction | 4.0 | 4.0 | 4.0 | 4.0 | 2.0 | 3.0 | 4.0 | 4.0 | 5.0 | 5.0 | 5.0 | 4.0 | 5.0 | 4.0 | 4.0 | 4.0 | 5.0 | 4.0 | 5.0 | 5.0 | 4.2 |
| M1: Hand & Peripheral Nerve | M1_Q3 | Carpal Instabilitites | 4.0 | 4.0 | 5.0 | 4.0 | 4.0 | 4.0 | 5.0 | 4.0 | 5.0 | 5.0 | 5.0 | 4.0 | 4.0 | 3.0 | 3.0 | 3.0 | 4.0 | 4.0 | 3.0 | 4.0 | 4.05 |
| M1: Hand & Peripheral Nerve | M1_Q4 | Ganglion Cyst | 4.0 | 4.0 | 5.0 | 4.0 | 5.0 | 5.0 | 5.0 | 4.0 | 5.0 | 5.0 | 4.0 | 5.0 | 4.0 | 4.0 | 4.0 | 4.0 | 3.0 | 3.0 | 3.0 | 4.0 | 4.2 |
| M1: Hand & Peripheral Nerve | M1_Q5 | Dupyutren | 4.0 | 4.0 | 2.0 | 3.0 | 4.0 | 4.0 | 3.0 | 4.0 | 2.0 | 2.0 | 5.0 | 5.0 | 5.0 | 5.0 | 5.0 | 5.0 | 4.0 | 4.0 | 5.0 | 5.0 | 4.0 |
| M1: Hand & Peripheral Nerve | M1_Q6 | SNAC | 4.0 | 4.0 | 4.0 | 4.0 | 2.0 | 2.0 | 3.0 | 4.0 | 5.0 | 5.0 | 4.0 | 4.0 | 4.0 | 4.0 | 4.0 | 4.0 | 4.0 | 4.0 | 5.0 | 5.0 | 3.95 |
| M1: Hand & Peripheral Nerve | M1_Q7 | Finger Movement | 4.0 | 4.0 | 3.0 | 3.0 | 4.0 | 4.0 | 4.0 | 4.0 | 5.0 | 5.0 | 4.0 | 4.0 | 4.0 | 4.0 | 4.0 | 5.0 | 3.0 | 4.0 | 5.0 | 5.0 | 4.1 |
| M1: Hand & Peripheral Nerve | M1_Q8 | DRUJ | 4.0 | 4.0 | 3.0 | 3.0 | 4.0 | 4.0 | 4.0 | 4.0 | 5.0 | 5.0 | 5.0 | 5.0 | 5.0 | 5.0 | 4.0 | 4.0 | 4.0 | 5.0 | 5.0 | 5.0 | 4.35 |
| M1: Hand & Peripheral Nerve | M1_Q9 | Compression points | 4.0 | 4.0 | 4.0 | 5.0 | 4.0 | 4.0 | 4.0 | 4.0 | 5.0 | 5.0 | 4.0 | 4.0 | 4.0 | 4.0 | 4.0 | 4.0 | 5.0 | 5.0 | 5.0 | 5.0 | 4.35 |
| M1: Hand & Peripheral Nerve | M1_Q10 | Pyogenic Flexor Tenosynovitis | 3.0 | 3.0 | 4.0 | 4.0 | 4.0 | 4.0 | 4.0 | 4.0 | 5.0 | 5.0 | 5.0 | 5.0 | 5.0 | 5.0 | 5.0 | 5.0 | 5.0 | 5.0 | 5.0 | 5.0 | 4.5 |
| M2: Reconstructive & Microsurgery | M2_Q1 | Mathes and Nahai | 1.0 | 1.0 | 4.0 | 4.0 | 4.0 | 4.0 | 4.0 | 4.0 | 4.0 | 4.0 | 4.0 | 2.0 | 4.0 | 1.0 | 4.0 | 1.0 | 4.0 | 2.0 | 4.0 | 1.0 | 3.05 |
| M2: Reconstructive & Microsurgery | M2_Q2 | STSG vs. FTSG | 5.0 | 5.0 | 5.0 | 5.0 | 5.0 | 5.0 | 5.0 | 5.0 | 5.0 | 5.0 | 4.0 | 4.0 | 4.0 | 4.0 | 4.0 | 4.0 | 5.0 | 5.0 | 4.0 | 4.0 | 4.6 |
| M2: Reconstructive & Microsurgery | M2_Q3 | Z-Plasty | 4.0 | 4.0 | 4.0 | 4.0 | 4.0 | 4.0 | 3.0 | 3.0 | 5.0 | 5.0 | 4.0 | 4.0 | 4.0 | 4.0 | 4.0 | 4.0 | 4.0 | 4.0 | 5.0 | 5.0 | 4.1 |
| M2: Reconstructive & Microsurgery | M2_Q4 | Random vs- Axial Pattern Flaps | 5.0 | 5.0 | 5.0 | 5.0 | 5.0 | 5.0 | 5.0 | 5.0 | 5.0 | 5.0 | 5.0 | 5.0 | 4.0 | 4.0 | 5.0 | 5.0 | 5.0 | 5.0 | 4.0 | 4.0 | 4.8 |
| M2: Reconstructive & Microsurgery | M2_Q5 | Random Pattern Flaps | 5.0 | 5.0 | 5.0 | 5.0 | 5.0 | 5.0 | 5.0 | 5.0 | 5.0 | 5.0 | 4.0 | 4.0 | 4.0 | 4.0 | 4.0 | 4.0 | 5.0 | 5.0 | 4.0 | 4.0 | 4.6 |
| M2: Reconstructive & Microsurgery | M2_Q6 | Medicinal Leeches | 5.0 | 5.0 | 5.0 | 5.0 | 5.0 | 5.0 | 5.0 | 5.0 | 5.0 | 5.0 | 5.0 | 5.0 | 5.0 | 5.0 | 5.0 | 5.0 | 5.0 | 5.0 | 5.0 | 5.0 | 5.0 |
| M2: Reconstructive & Microsurgery | M2_Q7 | Basics of the ALT flap | 4.0 | 4.0 | 5.0 | 5.0 | 5.0 | 5.0 | 4.0 | 4.0 | 5.0 | 5.0 | 5.0 | 5.0 | 5.0 | 5.0 | 4.0 | 4.0 | 4.0 | 4.0 | 4.0 | 4.0 | 4.5 |
| M2: Reconstructive & Microsurgery | M2_Q8 | Microsurgery Basics | 4.0 | 4.0 | 4.0 | 5.0 | 4.0 | 4.0 | 5.0 | 5.0 | 5.0 | 5.0 | 5.0 | 5.0 | 4.0 | 4.0 | 4.0 | 4.0 | 4.0 | 4.0 | 4.0 | 4.0 | 4.35 |
| M2: Reconstructive & Microsurgery | M2_Q9 | Partial Flap necrosis | 5.0 | 5.0 | 5.0 | 5.0 | 5.0 | 5.0 | 5.0 | 5.0 | 5.0 | 5.0 | 5.0 | 5.0 | 5.0 | 5.0 | 5.0 | 5.0 | 5.0 | 5.0 | 5.0 | 5.0 | 5.0 |
| M2: Reconstructive & Microsurgery | M2_Q10 | Ischemia Tolerance | 5.0 | 5.0 | 5.0 | 5.0 | 5.0 | 5.0 | 5.0 | 5.0 | 5.0 | 5.0 | 5.0 | 5.0 | 5.0 | 5.0 | 5.0 | 5.0 | 5.0 | 5.0 | 5.0 | 5.0 | 5.0 |
| M3: Burns & Critical Care | M3_Q1 | Burn center referral criteria | 1.0 | 2.0 | 4.0 | 4.0 | 4.0 | 4.0 | 4.0 | 4.0 | 5.0 | 5.0 | 5.0 | 5.0 | 4.0 | 5.0 | 5.0 | 5.0 | 5.0 | 5.0 | 5.0 | 5.0 | 4.3 |
| M3: Burns & Critical Care | M3_Q2 | ABSI | 5.0 | 5.0 | 5.0 | 5.0 | 5.0 | 5.0 | 5.0 | 5.0 | 5.0 | 5.0 | 5.0 | 5.0 | 5.0 | 5.0 | 5.0 | 5.0 | 5.0 | 5.0 | 5.0 | 5.0 | 5.0 |
| M3: Burns & Critical Care | M3_Q3 | Pathophysiology of burn shock | 5.0 | 5.0 | 5.0 | 5.0 | 5.0 | 5.0 | 5.0 | 5.0 | 5.0 | 5.0 | 5.0 | 5.0 | 5.0 | 5.0 | 4.0 | 5.0 | 4.0 | 4.0 | 4.0 | 4.0 | 4.75 |
| M3: Burns & Critical Care | M3_Q4 | Burn associated coagulopathy | 5.0 | 5.0 | 5.0 | 5.0 | 5.0 | 5.0 | 5.0 | 5.0 | 5.0 | 5.0 | 5.0 | 5.0 | 5.0 | 5.0 | 5.0 | 5.0 | 5.0 | 5.0 | 5.0 | 5.0 | 5.0 |
| M3: Burns & Critical Care | M3_Q5 | Hydrofluoric Acid Burn | 5.0 | 5.0 | 5.0 | 5.0 | 5.0 | 5.0 | 5.0 | 5.0 | 5.0 | 5.0 | 5.0 | 5.0 | 5.0 | 5.0 | 5.0 | 5.0 | 5.0 | 5.0 | 5.0 | 5.0 | 5.0 |
| M3: Burns & Critical Care | M3_Q6 | Volume Management | 5.0 | 5.0 | 5.0 | 5.0 | 5.0 | 5.0 | 5.0 | 5.0 | 5.0 | 5.0 | 5.0 | 5.0 | 5.0 | 5.0 | 5.0 | 5.0 | 5.0 | 5.0 | 5.0 | 4.0 | 4.95 |
| M3: Burns & Critical Care | M3_Q7 | Pathogens | 4.0 | 5.0 | 5.0 | 5.0 | 5.0 | 5.0 | 5.0 | 5.0 | 5.0 | 5.0 | 5.0 | 5.0 | 5.0 | 5.0 | 5.0 | 5.0 | 5.0 | 5.0 | 5.0 | 5.0 | 4.95 |
| M3: Burns & Critical Care | M3_Q8 | Carbon Monoxide Poisoning | 4.0 | 5.0 | 5.0 | 5.0 | 5.0 | 5.0 | 5.0 | 5.0 | 5.0 | 5.0 | 5.0 | 5.0 | 5.0 | 5.0 | 5.0 | 5.0 | 5.0 | 5.0 | 5.0 | 5.0 | 4.95 |
| M3: Burns & Critical Care | M3_Q9 | Epifascial excision | 2.0 | 4.0 | 5.0 | 5.0 | 5.0 | 5.0 | 5.0 | 5.0 | 2.0 | 3.0 | 5.0 | 5.0 | 5.0 | 5.0 | 4.0 | 4.0 | 3.0 | 3.0 | 2.0 | 2.0 | 3.95 |
| M3: Burns & Critical Care | M3_Q10 | Electrical Burn | 5.0 | 5.0 | 5.0 | 5.0 | 5.0 | 5.0 | 5.0 | 5.0 | 5.0 | 5.0 | 5.0 | 5.0 | 4.0 | 4.0 | 4.0 | 4.0 | 4.0 | 4.0 | 4.0 | 4.0 | 4.6 |
| M4: Aesthetic & Breast | M4_Q1 | IMF anatomy | 5.0 | 5.0 | 5.0 | 5.0 | 5.0 | 5.0 | 5.0 | 5.0 | 5.0 | 5.0 | 5.0 | 5.0 | 4.0 | 4.0 | 4.0 | 4.0 | 5.0 | 5.0 | 5.0 | 5.0 | 4.8 |
| M4: Aesthetic & Breast | M4_Q2 | Breast Ptosis | 5.0 | 5.0 | 5.0 | 5.0 | 5.0 | 5.0 | 5.0 | 5.0 | 5.0 | 5.0 | 5.0 | 5.0 | 5.0 | 5.0 | 5.0 | 5.0 | 5.0 | 5.0 | 5.0 | 5.0 | 5.0 |
| M4: Aesthetic & Breast | M4_Q3 | Impant Malposition | 5.0 | 5.0 | 5.0 | 5.0 | 5.0 | 5.0 | 5.0 | 5.0 | 5.0 | 5.0 | 4.0 | 4.0 | 4.0 | 4.0 | 4.0 | 4.0 | 5.0 | 5.0 | 5.0 | 5.0 | 4.7 |
| M4: Aesthetic & Breast | M4_Q4 | Baker classification | 4.0 | 4.0 | 5.0 | 5.0 | 5.0 | 5.0 | 5.0 | 5.0 | 5.0 | 5.0 | 5.0 | 5.0 | 5.0 | 5.0 | 5.0 | 5.0 | 5.0 | 5.0 | 5.0 | 5.0 | 4.9 |
| M4: Aesthetic & Breast | M4_Q5 | Complicaitons of Liposuction | 5.0 | 5.0 | 5.0 | 5.0 | 5.0 | 5.0 | 5.0 | 5.0 | 5.0 | 5.0 | 5.0 | 5.0 | 5.0 | 5.0 | 4.0 | 4.0 | 5.0 | 5.0 | 5.0 | 5.0 | 4.9 |
| M4: Aesthetic & Breast | M4_Q6 | Fate of transplanted fat | 4.0 | 5.0 | 5.0 | 5.0 | 5.0 | 5.0 | 5.0 | 5.0 | 5.0 | 5.0 | 4.0 | 4.0 | 4.0 | 4.0 | 4.0 | 4.0 | 5.0 | 5.0 | 5.0 | 5.0 | 4.65 |
| M4: Aesthetic & Breast | M4_Q7 | Processing of Lipoaspirate | 5.0 | 5.0 | 5.0 | 5.0 | 5.0 | 5.0 | 5.0 | 5.0 | 5.0 | 5.0 | 5.0 | 5.0 | 5.0 | 5.0 | 4.0 | 4.0 | 5.0 | 5.0 | 5.0 | 5.0 | 4.9 |
| M4: Aesthetic & Breast | M4_Q8 | Breast Anatomy | 4.0 | 4.0 | 5.0 | 5.0 | 5.0 | 5.0 | 5.0 | 5.0 | 5.0 | 5.0 | 4.0 | 4.0 | 4.0 | 4.0 | 4.0 | 4.0 | 3.0 | 4.0 | 4.0 | 4.0 | 4.35 |
| M4: Aesthetic & Breast | M4_Q9 | Vascular supply of the abdominal flap | 5.0 | 5.0 | 5.0 | 5.0 | 5.0 | 5.0 | 5.0 | 5.0 | 5.0 | 5.0 | 5.0 | 5.0 | 5.0 | 5.0 | 5.0 | 5.0 | 4.0 | 4.0 | 5.0 | 5.0 | 4.9 |
| M4: Aesthetic & Breast | M4_Q10 | Seroma formation after abdominoplasty | 5.0 | 5.0 | 5.0 | 5.0 | 5.0 | 5.0 | 5.0 | 5.0 | 5.0 | 5.0 | 4.0 | 4.0 | 5.0 | 5.0 | 4.0 | 5.0 | 5.0 | 5.0 | 4.0 | 3.0 | 4.7 |
| M5: Sarcoma & Oncology | M5_Q1 | Sarcoma Histology | 4.0 | 4.0 | 2.0 | 2.0 | 4.0 | 4.0 | 2.0 | 2.0 | 5.0 | 5.0 | 5.0 | 5.0 | 5.0 | 5.0 | 5.0 | 5.0 | 5.0 | 5.0 | 5.0 | 5.0 | 4.2 |
| M5: Sarcoma & Oncology | M5_Q2 | Prognostic Factors | 3.0 | 3.0 | 2.0 | 2.0 | 4.0 | 4.0 | 3.0 | 3.0 | 5.0 | 5.0 | 5.0 | 5.0 | 5.0 | 5.0 | 5.0 | 5.0 | 5.0 | 5.0 | 5.0 | 5.0 | 4.2 |
| M5: Sarcoma & Oncology | M5_Q3 | Management Protocols | 3.0 | 3.0 | 4.0 | 4.0 | 4.0 | 4.0 | 4.0 | 4.0 | 4.0 | 4.0 | 5.0 | 5.0 | 5.0 | 5.0 | 5.0 | 5.0 | 5.0 | 5.0 | 5.0 | 5.0 | 4.4 |
| M5: Sarcoma & Oncology | M5_Q4 | Radiotherapy | 4.0 | 4.0 | 1.0 | 1.0 | 4.0 | 4.0 | 1.0 | 1.0 | 5.0 | 5.0 | 4.0 | 4.0 | 4.0 | 4.0 | 5.0 | 5.0 | 4.0 | 4.0 | 5.0 | 5.0 | 3.7 |
| M5: Sarcoma & Oncology | M5_Q5 | Mimics | 5.0 | 5.0 | 5.0 | 5.0 | 5.0 | 5.0 | 5.0 | 5.0 | 5.0 | 5.0 | 5.0 | 5.0 | 5.0 | 5.0 | 5.0 | 5.0 | 5.0 | 5.0 | 4.0 | 4.0 | 4.9 |
| M5: Sarcoma & Oncology | M5_Q6 | Angiosarcoma | 5.0 | 5.0 | 5.0 | 5.0 | 5.0 | 5.0 | 5.0 | 5.0 | 5.0 | 5.0 | 5.0 | 5.0 | 5.0 | 5.0 | 5.0 | 5.0 | 5.0 | 5.0 | 5.0 | 5.0 | 5.0 |
| M5: Sarcoma & Oncology | M5_Q7 | Myxofibrosarcoma | 5.0 | 5.0 | 3.0 | 3.0 | 5.0 | 5.0 | 2.0 | 2.0 | 5.0 | 5.0 | 5.0 | 5.0 | 3.0 | 3.0 | 5.0 | 5.0 | 2.0 | 2.0 | 5.0 | 5.0 | 4.0 |
| M5: Sarcoma & Oncology | M5_Q8 | Merkel Cell | 5.0 | 5.0 | 4.0 | 4.0 | 5.0 | 5.0 | 4.0 | 4.0 | 5.0 | 5.0 | 5.0 | 5.0 | 4.0 | 4.0 | 4.0 | 4.0 | 5.0 | 5.0 | 5.0 | 5.0 | 4.6 |
| M5: Sarcoma & Oncology | M5_Q9 | Desmoid Tumor | 5.0 | 5.0 | 5.0 | 5.0 | 5.0 | 5.0 | 5.0 | 5.0 | 5.0 | 5.0 | 5.0 | 5.0 | 5.0 | 5.0 | 5.0 | 5.0 | 5.0 | 5.0 | 5.0 | 5.0 | 5.0 |
| M5: Sarcoma & Oncology | M5_Q10 | Clear cell sarcoma | 5.0 | 5.0 | 4.0 | 4.0 | 5.0 | 5.0 | 5.0 | 5.0 | 5.0 | 5.0 | 4.0 | 5.0 | 4.0 | 4.0 | 4.0 | 4.0 | 4.0 | 4.0 | 5.0 | 5.0 | 4.55 |
| M6: Core Surgical & Practice | M6_Q1 | ASS/Clopidogrel | 5.0 | 5.0 | 5.0 | 5.0 | 5.0 | 5.0 | 5.0 | 5.0 | 5.0 | 5.0 | 4.0 | 4.0 | 5.0 | 5.0 | 4.0 | 4.0 | 5.0 | 5.0 | 4.0 | 2.0 | 4.6 |
| M6: Core Surgical & Practice | M6_Q2 | Post abdominoplasty | 5.0 | 5.0 | 5.0 | 5.0 | 5.0 | 5.0 | 5.0 | 5.0 | 5.0 | 5.0 | 5.0 | 5.0 | 5.0 | 5.0 | 5.0 | 5.0 | 5.0 | 5.0 | 5.0 | 5.0 | 5.0 |
| M6: Core Surgical & Practice | M6_Q3 | Intraoperative Complication | 5.0 | 5.0 | 5.0 | 5.0 | 5.0 | 5.0 | 5.0 | 5.0 | 5.0 | 5.0 | 4.0 | 4.0 | 3.0 | 3.0 | 4.0 | 4.0 | 4.0 | 4.0 | 5.0 | 5.0 | 4.5 |
| M6: Core Surgical & Practice | M6_Q4 | TXA | 5.0 | 5.0 | 5.0 | 5.0 | 5.0 | 5.0 | 5.0 | 5.0 | 5.0 | 5.0 | 5.0 | 5.0 | 5.0 | 5.0 | 5.0 | 5.0 | 5.0 | 5.0 | 4.0 | 4.0 | 4.9 |
| M6: Core Surgical & Practice | M6_Q5 | Positioning | 5.0 | 5.0 | 4.0 | 4.0 | 5.0 | 5.0 | 5.0 | 5.0 | 5.0 | 5.0 | 5.0 | 5.0 | 5.0 | 5.0 | 4.0 | 5.0 | 5.0 | 5.0 | 5.0 | 5.0 | 4.85 |
| M6: Core Surgical & Practice | M6_Q6 | Nutritional Assessment | 5.0 | 5.0 | 5.0 | 5.0 | 5.0 | 5.0 | 5.0 | 5.0 | 5.0 | 5.0 | 5.0 | 5.0 | 5.0 | 5.0 | 5.0 | 5.0 | 5.0 | 5.0 | 5.0 | 5.0 | 5.0 |
| M6: Core Surgical & Practice | M6_Q7 | Bipolar | 4.0 | 4.0 | 4.0 | 4.0 | 5.0 | 5.0 | 5.0 | 5.0 | 5.0 | 5.0 | 4.0 | 4.0 | 4.0 | 4.0 | 5.0 | 5.0 | 4.0 | 4.0 | 4.0 | 4.0 | 4.4 |
| M6: Core Surgical & Practice | M6_Q8 | Drains | 1.0 | 1.0 | 4.0 | 4.0 | 4.0 | 4.0 | 4.0 | 4.0 | 5.0 | 5.0 | 4.0 | 4.0 | 5.0 | 5.0 | 4.0 | 4.0 | 4.0 | 4.0 | 5.0 | 5.0 | 4.0 |
| M6: Core Surgical & Practice | M6_Q9 | Smoking | 5.0 | 5.0 | 5.0 | 5.0 | 5.0 | 5.0 | 5.0 | 5.0 | 5.0 | 5.0 | 5.0 | 5.0 | 5.0 | 5.0 | 5.0 | 4.0 | 5.0 | 5.0 | 5.0 | 5.0 | 4.95 |
| M6: Core Surgical & Practice | M6_Q10 | HbA1c | 5.0 | 5.0 | 5.0 | 5.0 | 5.0 | 5.0 | 5.0 | 5.0 | 5.0 | 5.0 | 4.0 | 4.0 | 5.0 | 5.0 | 4.0 | 4.0 | 5.0 | 5.0 | 4.0 | 4.0 | 4.7 |

*Ratings by two independent faculty raters on a 5-point scale (1 = poor, 5 = excellent). Subscale ratings: Q = Question, E = Explanation. Dimensions: Accuracy = accuracy and currency; Relevance = relevance and objective alignment; Clarity = clarity and quality of construction; Pedagogical = pedagogical value; Safety = ethical and safety considerations. Mean Overall = grand mean across all dimensions and both raters.*

**Supplementary Table S3. Module-level performance, perception ratings, and knowledge outcomes.**

| **Module** | **N Completions** | **MCQ Accuracy Mean** | **MCQ Accuracy SD** | **MCQ Accuracy Median** | **MCQ Accuracy Range** | **Knowledge Gain Cohens d 95CI** | **Difficulty Rating Mean** | **Quality Rating Mean** | **Relevance Rating Mean** |
| --- | --- | --- | --- | --- | --- | --- | --- | --- | --- |
| Hand & Peripheral Nerve | 10 | 68.6 | 13.1 | 71.4 | 51.7-91.1 | 1.48 [0.71, 2.25] | 4.2 | 5.4 | 5.9 |
| Reconstructive & Microsurgery | 10 | 81.4 | 13.6 | 85.7 | 65.4-100.0 | 0.50 [-0.27, 1.27] | 4.7 | 5.3 | 5.6 |
| Burns & Critical Care | 10 | 58.6 | 25.6 | 50.0 | 11.6-88.4 | -0.23 [-1.00, 0.54] | 5.5 | 4.9 | 4.9 |
| Aesthetic & Breast | 10 | 78.6 | 15.4 | 78.6 | 55.4-100.0 | 0.93 [0.17, 1.70] | 4.8 | 5.4 | 5.7 |
| Sarcoma & Oncology | 11 | 55.8 | 30.9 | 42.9 | 0.0-89.3 | 0.14 [-0.63, 0.91] | 5.0 | 4.73 | 4.64 |
| Core Surgical & Practice | 9 | 92.1 | 7.5 | 85.7 | 85.7-100.0 | 0.00 [-0.77, 0.77] | 3.67 | 5.11 | 5.67 |
|  |  |  |  |  |  |  |  |  |  |
| Module-Level Correlations (Pearson r): |  |  |  |  |  |  |  |  |  |
| Difficulty Rating vs MCQ Accuracy | r = -0.753 |  |  |  |  |  |  |  |  |
| Quality Rating vs MCQ Accuracy | r = 0.592 |  |  |  |  |  |  |  |  |
| Relevance Rating vs MCQ Accuracy | r = 0.748 |  |  |  |  |  |  |  |  |
| Difficulty Rating vs Knowledge Gain | r = -0.310 |  |  |  |  |  |  |  |  |
| Quality Rating vs Knowledge Gain | r = 0.790 |  |  |  |  |  |  |  |  |
| Relevance Rating vs Knowledge Gain | r = 0.693 |  |  |  |  |  |  |  |  |

*MCQ = multiple-choice question. Knowledge change reported as Cohen’s d with 95% confidence interval (n = 9 matched pairs). Module-level correlations are based on n = 6 modules. Perception ratings are mean post-intervention resident ratings on 7-point Likert scales.*

**Supplementary Table S4. Complete parametric and non-parametric pre-post results for knowledge and confidence outcomes (n = 9 matched pairs).**

| **Topic** | **Measure** | **N Paired** | **Pre Median IQR** | **Post Median IQR** | **Median Diff** | **Pre Mean** | **Pre SD** | **Post Mean** | **Post SD** | **Mean Diff** | **Cohens d** | **CI 95** | **W** | **Wilcoxon p** | **r rb** | **Paired t p** | **MannWhitney p** | **Indep t p** | **N Pre** | **N Post** | **Shapiro p** |
| --- | --- | --- | --- | --- | --- | --- | --- | --- | --- | --- | --- | --- | --- | --- | --- | --- | --- | --- | --- | --- | --- |
| Composite (Overall) | Confidence | 9 | 4.17 (3.00-4.33) | 4.67 (4.17-5.17) | 0.33 | 3.94 | 1.04 | 4.46 | 0.92 | 0.52 | 0.65 | 0.65 (-0.11, 1.42) | 5.5 | 0.047* | 0.756 | 0.085 | 0.392 | 0.426 | 11 | 11 | 0.089 |
| Composite (Overall) | Knowledge | 9 | 4.00 (3.33-4.33) | 4.17 (4.00-4.83) | 0.17 | 4.04 | 0.9 | 4.37 | 0.71 | 0.33 | 0.71 | 0.71 (-0.06, 1.48) | 7.5 | 0.094 | 0.667 | 0.067 | 0.869 | 0.825 | 11 | 11 | 0.133 |
| M1: Hand & Peripheral Nerve | Confidence | 9 | 4 (4-5) | 5 (4-6) | 1.0 | 4.0 | 1.32 | 5.0 | 1.12 | 1.0 | 1.41 | 1.41 (0.64, 2.18) | 1.5 | 0.016* | 0.933 | 0.003* | 0.199 | 0.151 | 11 | 11 | 0.049 |
| M1: Hand & Peripheral Nerve | Knowledge | 9 | 4 (3-4) | 5 (4-5) | 1.0 | 3.67 | 1.12 | 4.56 | 0.88 | 0.89 | 1.48 | 1.48 (0.71, 2.25) | 1.5 | 0.016* | 0.933 | 0.002* | 0.346 | 0.211 | 11 | 11 | 0.012 |
| M2: Reconstructive & Microsurgery | Confidence | 9 | 4 (2-5) | 5 (3-5) | 0.0 | 3.78 | 1.92 | 4.22 | 1.64 | 0.44 | 0.39 | 0.39 (-0.38, 1.16) | 14.5 | 0.500 | 0.356 | 0.272 | 0.709 | 0.712 | 11 | 11 | 0.026 |
| M2: Reconstructive & Microsurgery | Knowledge | 9 | 4 (3-5) | 5 (4-5) | 0.0 | 4.11 | 1.45 | 4.56 | 1.24 | 0.44 | 0.5 | 0.50 (-0.26, 1.27) | 11.5 | 0.312 | 0.489 | 0.169 | 0.759 | 0.74 | 11 | 11 | 0.338 |
| M3: Burns & Critical Care | Confidence | 9 | 5 (4-5) | 5 (4-6) | 0.0 | 4.78 | 0.83 | 5.11 | 1.05 | 0.33 | 0.47 | 0.47 (-0.30, 1.24) | 12.0 | 0.375 | 0.467 | 0.195 | 0.81 | 0.69 | 11 | 11 | 0.024 |
| M3: Burns & Critical Care | Knowledge | 9 | 4 (4-6) | 4 (4-6) | 0.0 | 4.89 | 1.17 | 4.67 | 1.41 | -0.22 | -0.23 | -0.23 (-1.00, 0.54) | 18.0 | 0.750 | 0.2 | 0.512 | 0.33 | 0.384 | 11 | 11 | 0.273 |
| M4: Aesthetic & Breast | Confidence | 9 | 2 (2-3) | 3 (3-4) | 0.0 | 2.56 | 1.01 | 3.22 | 0.83 | 0.67 | 0.54 | 0.54 (-0.22, 1.31) | 11.0 | 0.250 | 0.511 | 0.141 | 0.273 | 0.297 | 11 | 11 | 0.286 |
| M4: Aesthetic & Breast | Knowledge | 9 | 2 (2-3) | 4 (3-4) | 1.0 | 2.56 | 0.73 | 3.33 | 0.87 | 0.78 | 0.93 | 0.93 (0.16, 1.70) | 5.0 | 0.062 | 0.778 | 0.023* | 0.09 | 0.115 | 11 | 11 | 0.025 |
| M5: Sarcoma & Oncology | Confidence | 9 | 4 (3-4) | 5 (3-5) | 1.0 | 3.78 | 0.97 | 4.33 | 1.12 | 0.56 | 0.45 | 0.45 (-0.32, 1.22) | 12.5 | 0.359 | 0.444 | 0.214 | 0.426 | 0.418 | 11 | 11 | 0.195 |
| M5: Sarcoma & Oncology | Knowledge | 9 | 4 (4-4) | 4 (4-5) | 0.0 | 4.22 | 1.09 | 4.33 | 0.71 | 0.11 | 0.14 | 0.14 (-0.63, 0.91) | 19.0 | 1.000 | 0.156 | 0.681 | 0.887 | 0.648 | 11 | 11 | 0.055 |
| M6: Core Surgical & Practice | Confidence | 9 | 5 (4-6) | 5 (5-5) | 0.0 | 4.78 | 1.39 | 4.89 | 0.93 | 0.11 | 0.11 | 0.11 (-0.66, 0.87) | 19.0 | 1.000 | 0.156 | 0.760 | 0.973 | 1.0 | 11 | 11 | 0.025 |
| M6: Core Surgical & Practice | Knowledge | 9 | 5 (4-6) | 5 (4-5) | 0.0 | 4.78 | 1.39 | 4.78 | 0.67 | 0.0 | 0.0 | 0.00 (-0.77, 0.77) | 20.0 | 0.938 | 0.111 | 1.000 | 0.537 | 0.544 | 11 | 11 | 0.239 |

** P < .05. W = Wilcoxon signed-rank test statistic; r = rank-biserial correlation (nonparametric effect size); d = Cohen’s d with 95% confidence interval. Shapiro P = Shapiro-Wilk normality test on paired differences. Two outcomes were discordant between Wilcoxon and paired t-test: Composite Confidence (Wilcoxon P = .047 vs t-test P = .085) and Aesthetic Knowledge (Wilcoxon P = .063 vs t-test P = .023).*

**Supplementary Table S5. Pre-post changes in secondary outcome measures.**

| **Category** | **Item** | **Timepoint** | **n** | **Mean** | **SD** | **Median** | **Min** | **Max** | **Mean Difference** | **Cohens d** | **Wilcoxon p** | **W** | **r** |
| --- | --- | --- | --- | --- | --- | --- | --- | --- | --- | --- | --- | --- | --- |
| Resource Utilization |  |  |  |  |  |  |  |  |  |  |  |  |  |
|  | Textbook Use | Pre | 8.0 | 4.0 | 0.53 | 4.0 | 3.0 | 5.0 | 0.12 | 0.35 | 1.0 | 14.0 | 0.222 |
|  |  | Post | 8.0 | 4.12 | 0.35 | 4.0 | 4.0 | 5.0 |  |  |  |  |  |
|  | Journal Use | Pre | 9.0 | 3.22 | 0.83 | 3.0 | 2.0 | 4.0 | 0.44 | 0.61 | 0.25 | 10.5 | 0.533 |
|  |  | Post | 9.0 | 3.67 | 0.5 | 4.0 | 3.0 | 4.0 |  |  |  |  |  |
|  | Departmental Materials | Pre | 9.0 | 4.56 | 0.53 | 5.0 | 1.0 | 7.0 | -0.33 | -0.67 | 0.25 | 10.5 | 0.533 |
|  |  | Post | 9.0 | 4.22 | 0.44 | 4.0 | 1.0 | 7.0 |  |  |  |  |  |
|  | YouTube Videos | Pre | 9.0 | 3.67 | 0.5 | 4.0 | 3.0 | 4.0 | 0.11 | 0.18 | 1.0 | 18.5 | 0.178 |
|  |  | Post | 9.0 | 3.78 | 0.83 | 4.0 | 2.0 | 5.0 |  |  |  |  |  |
|  | Database Use | Pre | 8.0 | 4.12 | 0.35 | 4.0 | 4.0 | 5.0 | -0.25 | -0.35 | 0.625 | 11.5 | 0.361 |
|  |  | Post | 8.0 | 3.88 | 0.64 | 4.0 | 3.0 | 5.0 |  |  |  |  |  |
|  | AI Tool Use | Pre | 9.0 | 3.67 | 1.12 | 4.0 | 1.0 | 5.0 | 0.22 | 0.2 | 0.625 | 16.0 | 0.289 |
|  |  | Post | 9.0 | 3.89 | 0.93 | 4.0 | 1.0 | 5.0 |  |  |  |  |  |
| Information Seeking |  |  |  |  |  |  |  |  |  |  |  |  |  |
|  | Information Seeking Behavior | Pre | 9.0 | 5.44 | 0.73 | 6.0 | 4.0 | 6.0 | 0.11 | 0.14 | 1.0 | 19.0 | 0.156 |
|  |  | Post | 9.0 | 5.56 | 1.24 | 6.0 | 3.0 | 7.0 |  |  |  |  |  |
| AI Familiarity |  |  |  |  |  |  |  |  |  |  |  |  |  |
|  | AI Familiarity & Comfort | Pre | 9.0 | 3.0 | 1.66 | 3.0 | 1.0 | 5.0 | 0.89 | 0.7 | 0.125 | 8.0 | 0.644 |
|  |  | Post | 9.0 | 3.89 | 1.45 | 4.0 | 1.0 | 5.0 |  |  |  |  |  |
| Sense of Community |  |  |  |  |  |  |  |  |  |  |  |  |  |
|  | Program Addressing Shared Needs | Pre | 9.0 | 5.11 | 1.17 | 6.0 | 3.0 | 6.0 | 0.0 | 0.0 | 1.0 | 20.5 | 0.089 |
|  |  | Post | 9.0 | 5.11 | 1.17 | 5.0 | 3.0 | 7.0 |  |  |  |  |  |
|  | Peer Discussion | Pre | 9.0 | 5.67 | 0.71 | 6.0 | 3.0 | 7.0 | -0.11 | -0.18 | 1.0 | 18.5 | 0.178 |
|  |  | Post | 9.0 | 5.56 | 0.88 | 6.0 | 3.0 | 7.0 |  |  |  |  |  |
|  | Connection to Peers | Pre | 9.0 | 5.22 | 0.97 | 5.0 | 3.0 | 6.0 | 0.33 | 0.47 | 0.5 | 14.0 | 0.378 |
|  |  | Post | 9.0 | 5.56 | 1.24 | 6.0 | 3.0 | 7.0 |  |  |  |  |  |
|  | Psychological Safety | Pre | 9.0 | 5.56 | 1.33 | 5.0 | 3.0 | 7.0 | 0.11 | 0.11 | 1.0 | 21.0 | 0.067 |
|  |  | Post | 9.0 | 5.67 | 1.0 | 6.0 | 4.0 | 7.0 |  |  |  |  |  |
|  | Peer Support | Pre | 9.0 | 5.11 | 1.17 | 5.0 | 3.0 | 7.0 | -0.11 | -0.12 | 1.0 | 21.5 | 0.044 |
|  |  | Post | 9.0 | 5.0 | 1.41 | 5.0 | 3.0 | 7.0 |  |  |  |  |  |

*Resource utilization items rated on a 5-point frequency scale (1 = never to 5 = daily). AI familiarity rated on a 7-point Likert scale (1 = not at all familiar to 7 = very familiar). Sense of community items rated on a 7-point Likert scale (1 = strongly disagree to 7 = strongly agree). W = Wilcoxon signed-rank test statistic; r = rank-biserial correlation.*
